# Loose coupling between Ca^2+^ channels and release sensors as a synaptic correlate of higher-order brain function

**DOI:** 10.7554/eLife.110314

**Published:** 2026-10-28

**Authors:** Grit Bornschein, Max Schwarze, Antonia Brunner, Akanksha Arshia, Simone Brachtendorf, Hartmut Schmidt

**Affiliations:** 1 Carl Ludwig Institute for Physiology, Medical Faculty, University of Leipzig Leipzig Germany; University at Albany, State University of New York United States; Stanford University School of Medicine United States

**Keywords:** prefrontal cortex, pyramidal neurons, coupling distance, short-term plasticity, Mouse

## Abstract

In the mature neocortex, functionally distinct areas are built by the same archetypes of neurons, but depending on the area, these neurons and their synapses are engaged in very different functions, ranging from lower-order processing of sensory information to higher-order associations and cognitive functions. We found significant differences in the functional presynaptic nanoarchitectures of the same types of pyramidal neuron synapses, depending on whether they are located in the prefrontal cortex (PFC) or in the primary somatosensory cortex (S1) of mice. Mature synapses in PFC operated with loose microdomain coupling, as opposed to tight nanodomain coupling in S1. These differences were associated with significant differences in synaptic timing, efficacy, and plasticity between areas. Our data suggest that the mature neocortex uses tuning of synaptic topographies to specialize seemingly identical types of neurons for their required function. They suggest that microdomain coupling in pyramidal neuron synapses could be a presynaptic structure-function correlate of higher-order neocortical functions.

## Introduction

The neo- or isocortex is a morphologically homogeneous brain region that covers highly diverse functions within its specialized areas, ranging from early sensory processing and motor control up to higher-order associations, cognition, and consciousness (Douglas and Martin, 2004; Harris and Shepherd, 2015). Notably, all of these diverse functions are executed by the same archetypes of neurons and synapses. However, depending on the area, they are engaged to different degrees in lower- or higher-order processing. Whether area-specific functional differences in the mature neocortex are associated with or even arise from differences in the functional presynaptic nanoarchitecture of the same principal types of synapses is currently unclear.

At presynaptic active zones, the coupling distance between voltage-gated Ca^2+^ channels (VGCCs) and transmitter-filled synaptic vesicles (SVs) is a major determinant of key synaptic properties, including speed, efficacy, and reliability of synaptic transmission (Rozov et al., 2001; Fedchyshyn and Wang, 2005; Bucurenciu et al., 2008; Baur et al., 2015; Bornschein et al., 2019a; Chen et al., 2024), and also of short-term plasticity, although the latter can be obscured by vesicle replenishment (Miki et al., 2016; Doussau et al., 2017; Bornschein et al., 2019b; Lin et al., 2022). Studies at different synapses in different parts of the brain suggest that excitatory synapses engaged in reliable information transfer switch from loose microdomain to tight nanodomain coupling during development (Fedchyshyn and Wang, 2005; Baur et al., 2015; Nakamura et al., 2015; Bornschein et al., 2019a). This includes synapses between layer 5 pyramidal neurons (L5PNs) in the primary somatosensory cortex (S1), an area of early sensory processing (Bornschein et al., 2019a). On the other hand, loose microdomain coupling was found in the mature brain to date only at a highly plastic hippocampal synapse and has been suggested to provide a molecular framework for presynaptic plasticity (Vyleta and Jonas, 2014). However, if microdomain coupling also represents a synaptic correlate of higher-order neocortical function or if it is restricted to specific and highly plastic synapses in the hippocampus is currently unclear. Therefore, here we tested the hypothesis that loose microdomain coupling is the synaptic correlate of higher-order functions of the mature neocortex, whereas tight nanodomain coupling is the synaptic correlate of earlier processing stages. Our data show that in the mature cortex the same types of pyramidal neuron synapses operate with loose microdomain coupling if they are located in the prefrontal cortex (PFC), whereas in S1 they operate with tight nanodomain coupling. Thus, our results corroborate the hypothesis and suggest microdomain coupling as a synaptic correlate of higher-order function in the neocortex.

## Results

### Synaptic efficacy, reliability, and short-term plasticity differ between PFC and S1

**Figure 1.**
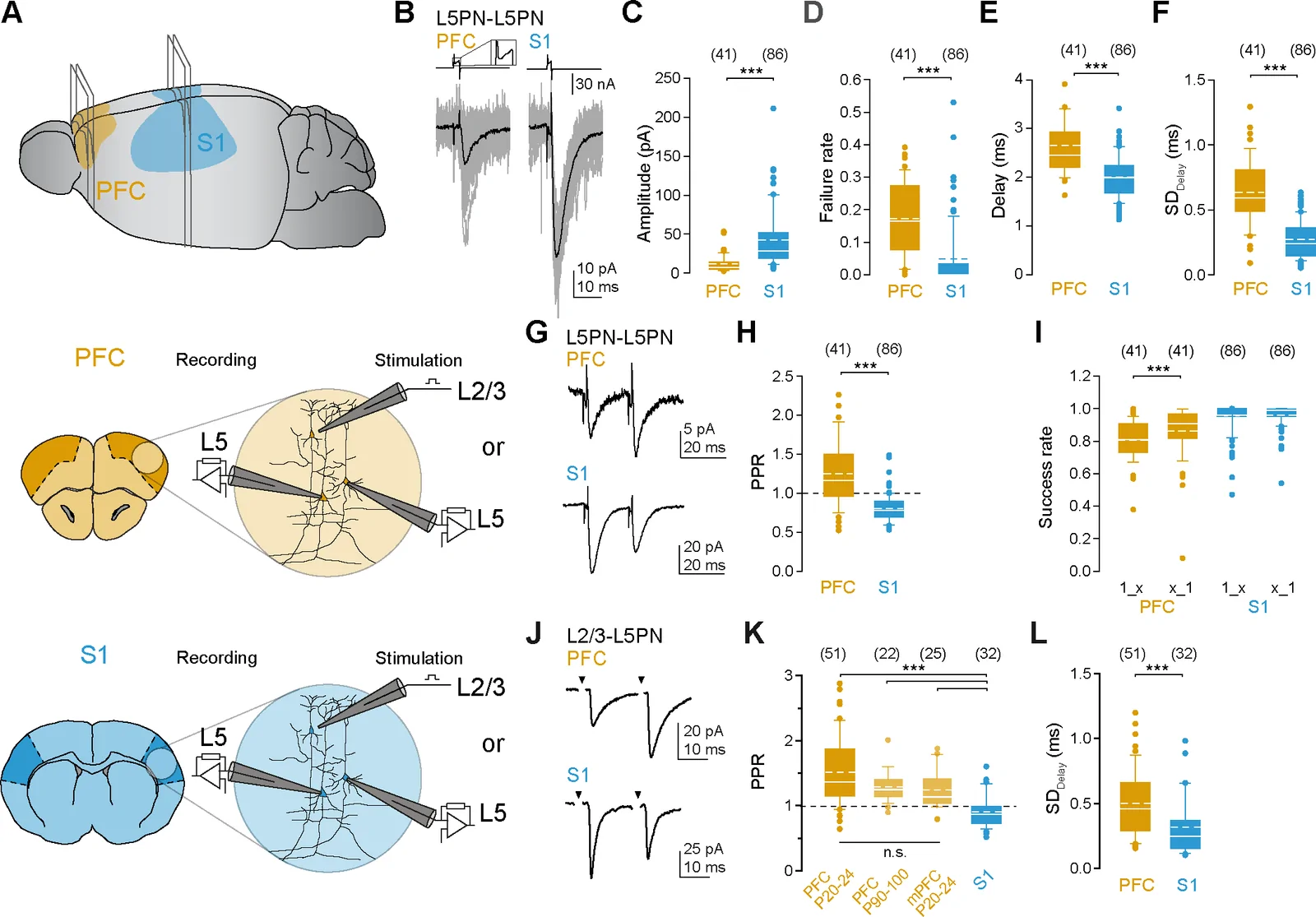
Differences in synaptic transmission and plasticity between prefrontal cortex (PFC) and primary somatosensory cortex (S1) synapses. (**A**) Schematic representation of the mature mouse brain (top) and the positions at which coronal sections were made from PFC (orange; middle) or S1 (blue; bottom). Recordings were made in acute slices from layer 5 pyramidal neuron (L5PN)-L5PN pairs or from L2/3-L5PN connections. (**B**) EPSCs (bottom) recorded from postsynaptic L5PNs after evoking action currents in the presynaptic L5PNs (top, inset: threefold magnification) in PFC (left) or S1 (right), individual recordings in gray, averages in black. (**C–F**) Summary of basic synaptic properties: EPSC amplitudes (**C**), failure rates (**D**), synaptic delays (**E**), and their SD values (**F**) in PFC and S1 (medians ± IQRs, means as dashed lines, whiskers represent 10th and 90th percentiles, dots indicate outliers, numbers of cell pairs in brackets; ***p<0.001, Mann-Whitney U test [MWU]). Data from S1 are from Bornschein et al., 2019a. (**G**) EPSCs recorded from pairs of L5PNs at 50 Hz intervals in PFC (top) and S1 (bottom), presynaptic stimulations are omitted, averages from at least 10 individual recordings. (**H, I**) Summary of paired pulse ratios (PPRs) (**H**; ***p<0.001, MWU) and success rates (**I**) following the 1st (1_x) and second stimulation (x_1; ***p<0.001, p=0.987, Wilcoxon signed-rank test [WSR]). S1 data are from Bornschein et al., 2019b. (**J**) As in (**G**) but for L2/3-L5 synapses (bottom, averages from at least 15 individual recordings, stimulation artifacts were blanked for clarity, arrowheads denote the time points of extracellular stimulation). (**K**) Boxplots summarizing PPR data from lateral PFC (PFC), medial PFC (mPFC), older PFC (P90–100), and S1 (***p<0.001, n.s., not significant, ANOVA on ranks). (**L**) Comparison of SDs of the synaptic delays at L2/3-L5 synapses between PFC and S1 (***p<0.001, MWU). Figure 1—source data 1.Data from recordings of L5PN-L5PN connections corresponding to Figure 1B-I. Figure 1—source data 2.Data from recordings of L2/3-L5PN connections corresponding to Figure 1J-L.

We focused on the lateral PFC and medial prefrontal cortex (mPFC) as areas of higher-order processing and on S1 as an area of lower-order processing. Within these areas we investigated excitatory synapses between pyramidal neurons (PNs), which are the principal building blocks of the neocortex. We first analyzed the properties of synapses connecting neighboring L5PNs in mature PFC (P21–26) by performing paired whole-cell patch-clamp recordings and compared their properties to those we previously reported for the same synapses and age in S1 (Bornschein et al., 2019b; Bornschein et al., 2019a; Figure 1A, Table 1). S1 data from both previous studies were combined, and additional analyses of synaptic delays, paired pulse ratios (PPRs), and success rates were performed. We found that the efficacy of transmission, i.e., the size of a unitary EPSC evoked by a single action potential (Rozov et al., 2001), was significantly lower in PFC (8 pA, 5–15 pA) than in S1 (29 pA, 18–53 pA), whereas the synaptic failure rates were significantly higher in PFC (0.16, 0.08–0.28) than in S1 (0, 0–0.03; Figure 1B–D). In addition, we found that the synaptic delays (2.46 ms, 2.20–2.94 ms), as well as their standard deviation (SD_Delay_: 0.60 ms, 0.49–0.81 ms), were significantly larger in PFC than in S1 (delay: 1.99 ms, 1.67–2.25 ms; SD_Delay_: 0.24 ms, 1.14–0.37 ms; Figure 1E and F), indicating that transmitter release is less tightly coupled to the time of the action potential in PFC than in S1 (Bullmann et al., 2024). Together, these data show that synaptic speed, efficacy, and reliability during single action potentials are lower in PFC than at the same synapses in S1.

**Figure 1—figure supplement 1.**
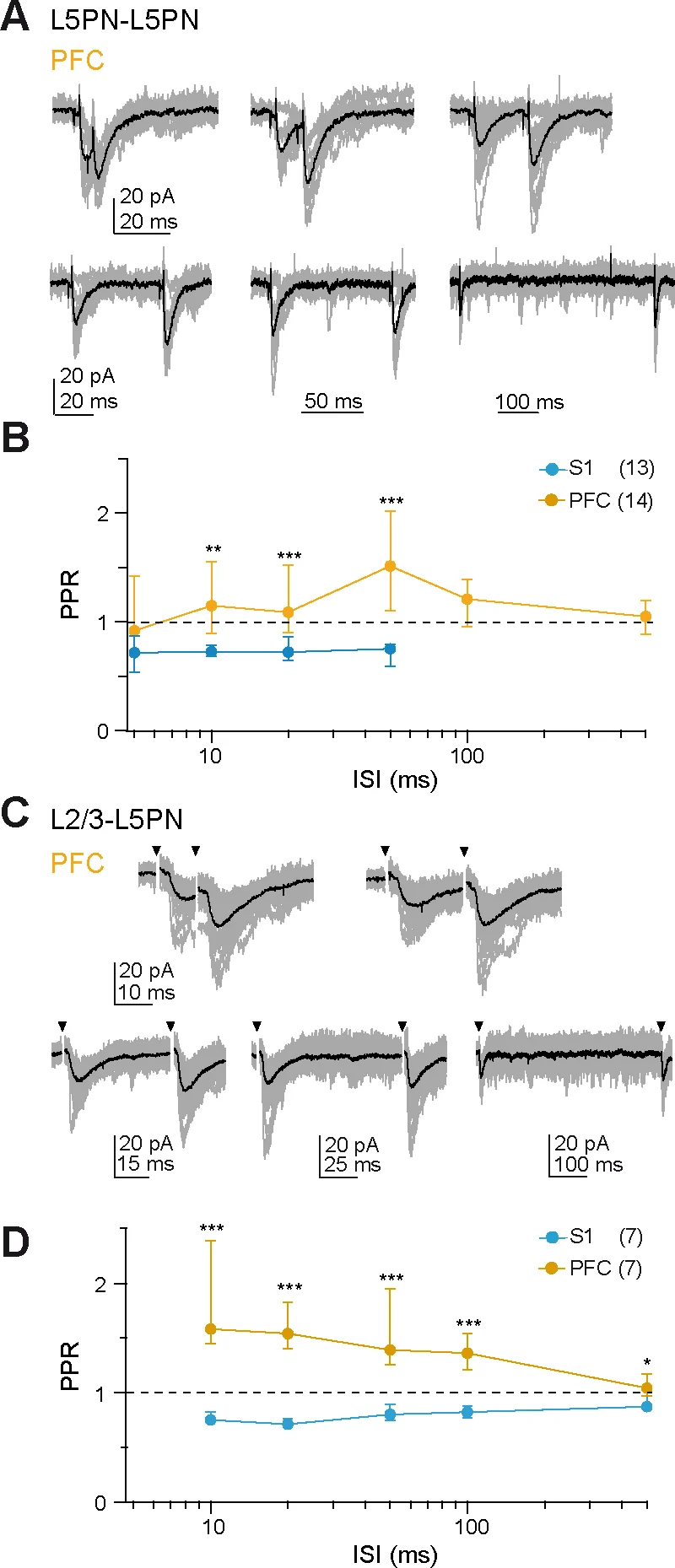
Short-term plasticity in prefrontal cortex (PFC) and primary somatosensory cortex (S1). (**A**) EPSCs recorded from a layer 5 pyramidal neuron (L5PN)-L5PN pair in PFC at interstimulus intervals (ISIs) ranging from 5 to 500 ms. (**B**) Comparison of paired pulse ratios (PPRs) recorded at different ISIs from pairs of L5PNs in PFC (orange) and S1 (blue). Averages shown as medians and interquartile ranges (IQRs) (n in brackets; p=0.104, **p=0.008, ***p<0.001, Mann-Whitney U test [MWU]). S1 data are from Bornschein et al., 2019b. (**C**) Same as in (**A**), but for recordings from prefrontal L5PNs after extracellular stimulation in L2/3 with paired pulses at ISIs of 10 to 500 ms. (**D**) Same as in (**B**), but for L2/3-L5PN synapses in PFC and S1 (***p<0.001, MWU). Figure 1—figure supplement 1—source data 1.Data from recordings of L5PN-L5PN connections corresponding to Figure 1—figure supplement 1B. Figure 1—figure supplement 1—source data 2.Data from recordings of L2/3-L5PN connections corresponding to Figure 1—figure supplement 1D.

**Figure 1—figure supplement 2.**
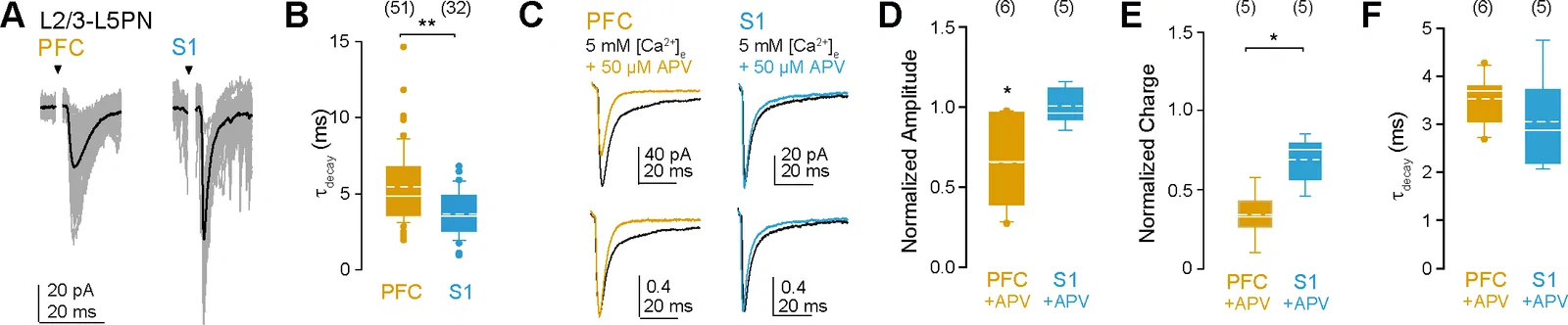
Different NMDA receptor contributions in prefrontal cortex (PFC) and primary somatosensory cortex (S1). (**A**) Examples of EPSCs recorded from L2/3-layer 5 pyramidal neuron (L5PN) connections in PFC and S1. (**B**) Summary of EPSC decay time constants (*τ*_decay_) in PFC and S1 (**p=0.002, Mann-Whitney U test [MWU]). (**C**) Top: Averaged example recordings before (baseline in black) and after application of 50 μM 2-amino-5-phosphonovaleric acid (APV) in PFC (orange) and S1 (blue). Bottom: EPSC recordings from top graphs normalized to baseline emphasizing changes in synaptic decay. (**D–F**) Box plots comparing normalized EPSC amplitudes (**D**; *p=0.031, p=1.0, Wilcoxon signed-rank test [WSR]), their charges (**E**; *p=0.016, MWU), and *τ*_decay_ values in the presence of 50 µM APV (**F**; p=0.329) in PFC and S1. Figure 1—figure supplement 2—source data 1.Data from recordings of L2/3-L5PN connections corresponding to Figure 1—figure supplement 2B-F.

**Table 1.** New and previously published data.

Synapse/region	Experiments/analyses	Figures	Data
L5PN-L5PN PFC	EPSC amplitudes, failure rates, delays, SD_Delay_, PPRs, success rates	Figure 1B–I Figure 1—figure supplement 1A and B	This study
L5PN-L5PN S1	EPSC amplitudes, failure rates; additional quantification of delays, SD_Delay_s, PPRs, success rates	Figure 1B–I Figure 1—figure supplement 1B	Bornschein et al., 2019b; Bornschein et al., 2019a
L2/3-L5PN PFC and S1	PPRs, SD_Delay_	Figure 1J–L Figure 1—figure supplement 1C and D	This study
L2/3-L5PN PFC and S1	NMDA receptor contribution	Figure 1—figure supplement 2A–F	This study
L5PN-L5PN PFC	Chelator experiments, CD	Figure 2A and B	This study
L5PN-L5PN S1	Chelator experiments, CD; Reanalyses of EGTA effects at 20–30 min test period	Figure 2B	Bornschein et al., 2019a
L2/3-L5PN PFC and S1	Chelator experiments, CD	Figure 2C–F Figure 2—figure supplement 1A–E	This study
L5PN-L5PN PFC	MPFA	Figure 3A, D, and E	This study
L5PN-L5PN S1	MPFA	Figure 3D and E	Bornschein et al., 2019a
L2/3-L5PN PFC and S1	MPFA	Figure 3B, C, and F	This study
L5PN PFC	2P Ca^2+^ imaging	Figure 4A–F	This study
L5PN S1	2P Ca^2+^ imaging	Figure 4A–F Figure 4—figure supplement 1A	This study
L5PN S1	2P Ca^2+^ imaging: basal [Ca^2+^]_i_ with Fluo-5F and OGB-1	Figure 4—figure supplement 1B	Bornschein et al., 2025; this study
L2/3PN and L5PN PFC	Modeling	Figure 5A–D Figure 5—figure supplement 1	This study
L2/3-L5PN PFC	Cd^2+^ experiments	Figure 5E and F	This study
L2/3-L5PN PFC	Cd^2+^ experiments	Figure 5E and F	This study

When pairs of action potentials were applied at high frequency (Figure 1G–I), differences in the synaptic success rates were associated with a significant difference in the PPR between PFC and S1. In PFC paired pulse facilitation (PPF, 1.18, 0.98–1.52) prevailed associated with increased success rates in the second pulse (1_x: 0.81, 0.73–0.91; x_1: 0.91, 0.82–0.97). In contrast, in S1 paired pulse depression (PPD, 0.78, 0.69–0.90) was found with high initial success rates (1_x: 1.00, 0.97–1.00; x_1: 0.99, 0.95–1.00). We observed the difference in short-term plasticity over a range of interstimulus intervals (ISIs) of 5–50 ms (Figure 1—figure supplement 1A and B). Thus, the differences in synaptic efficacy and reliability between S1 and PFC during the first action potential correlate with differential short-term plasticity, with the emphasis in S1 being on the first transmission process, while in PFC, facilitation emphasizes the subsequent release.

Next, we investigated transmission at synapses between L2/3 and L5PNs, using minimal extracellular stimulation near somata of L2/3PNs (Figure 1A). We also found that L2/3-L5PN synapses in PFC showed PPF (1.36, 1.14–1.88) as opposed to PPD in S1 (0.87, 0.72–0.99; Figure 1J and K). We observed this over a range of ISIs up to 100 ms (Figure 1—figure supplement 1C and D). In addition, at PFC synapses, the SD of the synaptic delays (0.46, 0.29–0.66) was again significantly larger than in S1 (0.25, 0.15–0.37; Figure 1L). Furthermore, we found larger EPSC decay time constants (*τ*_decay_) in PFC compared to S1, possibly reflecting a contribution of different postsynaptic glutamate receptors (Figure 1—figure supplement 2A and B). Indeed, application of the N-methyl-D-aspartate receptor (NMDAR) antagonist 2-amino-5-phosphonovaleric acid (APV) removed the difference in *τ*_decay_ between PFC and S1 (Figure 1—figure supplement 2C–F). We extended the paired pulse experiments at L2/3-L5PN synapses in PFC to even older mice (P90–100) and to the mPFC (P21–26). We found that PPF persisted in the older age window (1.25, 1.14–1.41) and that also the synapses in mPFC showed PPF (1.14, 1.03–1.42; Figure 1K).

Together, these results from glutamatergic synapses onto L5PNs show significant differences in the synaptic delays, efficacy, reliability, and short-term plasticity between PFC and S1. The results would be consistent with area-specific differences in the functional coupling distances between VGCCs and SVs at the presynaptic active zones.

### EGTA sensitivity of release is higher in PFC than in S1

**Figure 2.**
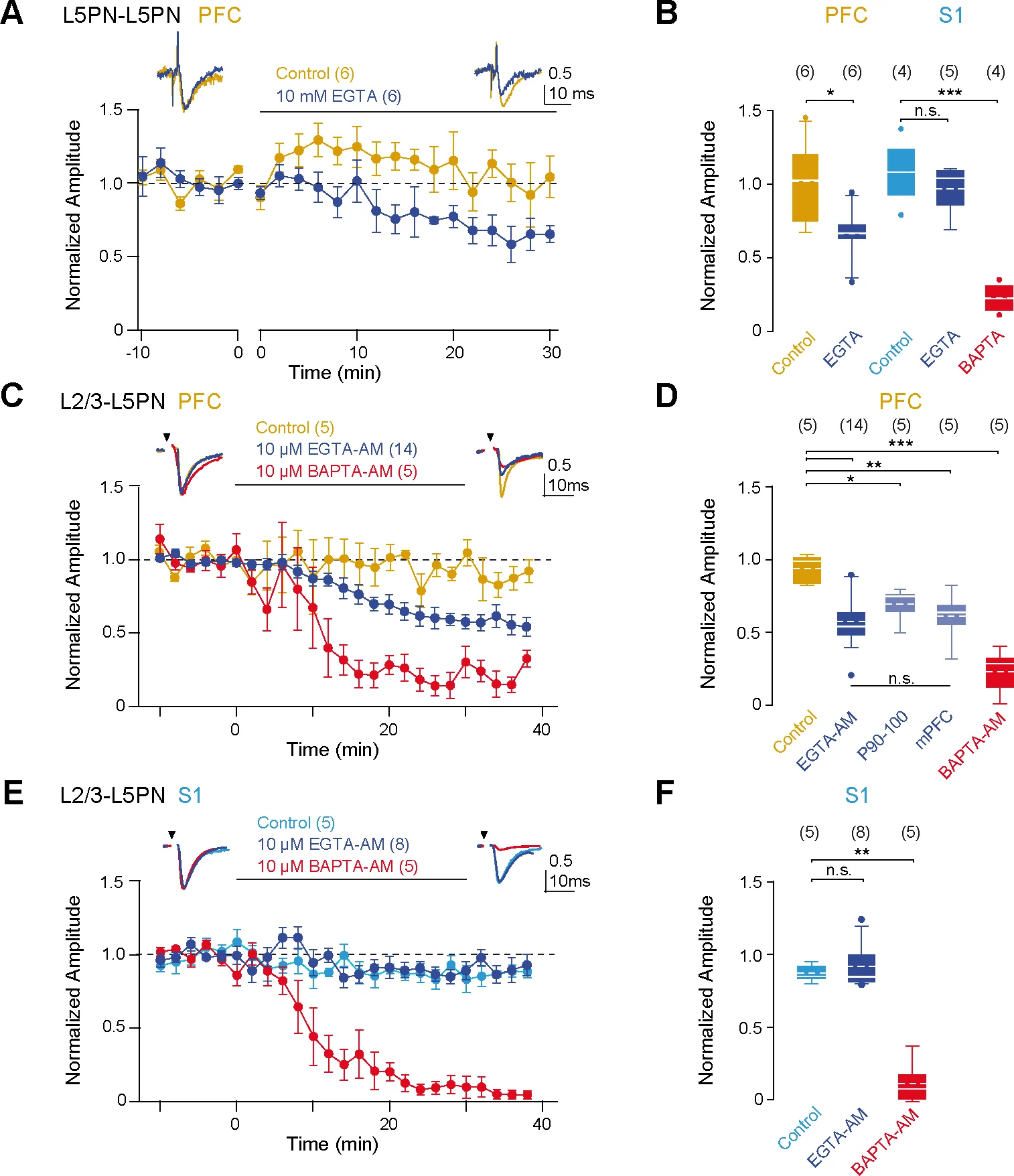
Area-specific differences in EGTA sensitivity. (**A**) Averaged, baseline-normalized EPSC amplitudes (means ± SEMs, 2 min bins) recorded from layer 5 pyramidal neuron (L5PN)-L5PN pairs in prefrontal cortex (PFC) with standard pipette solution (control, orange) and after re-patching the presynaptic neuron with a pipette solution containing 10 mM EGTA (blue). Insets: Average EPSCs from one pair during baseline (−10 to 0 min) and test period (20–30 min) for control and EGTA (averages normalized to baseline). (**B**) Boxplot comparing the normalized EPSC amplitudes prior to and following the application of EGTA and/or BAPTA with control recordings in L5PN pairs in PFC (*p=0.041, Mann-Whitney U test [MWU]) and primary somatosensory cortex (S1) (***p<0.001, p=0.365, one-way ANOVA; Shapiro-Wilk normality test: p=0.597). S1 data are from Bornschein et al., 2019a. Note the significant EGTA effect. (**C**) As in (**A**) but for L2/3-L5PN connections in PFC. Following baseline recordings (10 min), slices were perfused for 30 min (solid line) with artificial cerebrospinal fluid (ACSF) containing either 10 μM EGTA-AM (blue), 10 μM BAPTA-AM (red), or only the solvent DMSO/Pluronic (control, orange), and thereafter rinsed for 10 min with ACSF (test period). Insets: Averaged EPSCs during baseline and test period. (**D**) Summary of chelator-AM effects in PFC, medial PFC (mPFC), and older PFC (P90–100; ***p<0.001, **p=0.009, *p=0.032, one-way ANOVA vs. control; Shapiro-Wilk normality test: p=0.666). Note the similar EGTA sensitivity of release in all PFC recordings (p=0.347, one-way ANOVA). (**E**) As in (**C**) but for L2/3-L5PN connections in S1 (control in light blue). (**F**) Summary of chelator-AM effects in S1 (**p=0.006, ANOVA on ranks). Note the absence of a significant EGTA effect in S1 (p=0.943, MWU). Figure 2—source data 1.Data from recordings of L5PN-L5PN connections corresponding to Figure 2A and B. Figure 2—source data 2.Data from recordings of L2/3-L5PN connections corresponding to Figure 2C-F, Figure 2—figure supplement 1.

To directly test for differences in the coupling distances, we investigated the sensitivity of release to synthetic Ca^2+^ chelators (Figure 2). The sensitivity of release to low millimolar concentrations of the kinetically slow Ca^2+^ chelator EGTA is a standard indicator of loose coupling (Eggermann et al., 2012). First, we performed recordings at pairs of L5PNs in mature PFC (Figure 2A and B). After 10 min of stable baseline recordings, the presynaptic neuron was patched a second time and equilibrated for 30 min with 10 mM EGTA-containing pipette solution in the whole-cell configuration. In these paired recordings, we found a significant effect of EGTA on release in PFC (0.67, 0.63–0.73). In control recordings, a temporary initial increase in EPSC amplitudes after whole-cell access to the presynaptic neuron was evident. Although L5PNs do not express large concentrations of mobile buffers (Helmchen et al., 1996; Tran and Stricker, 2018; Bornschein et al., 2019a), their wash-out might have contributed to the temporary run-up. On the other hand, run-up correlated with a temporary decline in R_s_ in some recordings (3 out of 6). Run-up effects normalized after 20 min at the latest (1.02, 0.75–1.20). In identical experiments in S1, we had previously found release to be insensitive to 10 mM EGTA (1.04, 0.80–1.10; control: 1.08, 0.86–1.31) (Bornschein et al., 2019a). For comparison, data from the previous study were reanalyzed for the test period between 20 and 30 min. EPSC amplitudes in S1 were sensitive to the same concentration of the kinetically fast Ca^2+^ chelator BAPTA (test period 10–20 min: 0.22, 0.13–0.33). These data provide evidence that the synapses between L5PNs in mature PFC do indeed operate with loose coupling, in contrast to their counterparts in S1.

**Figure 2—figure supplement 1.**
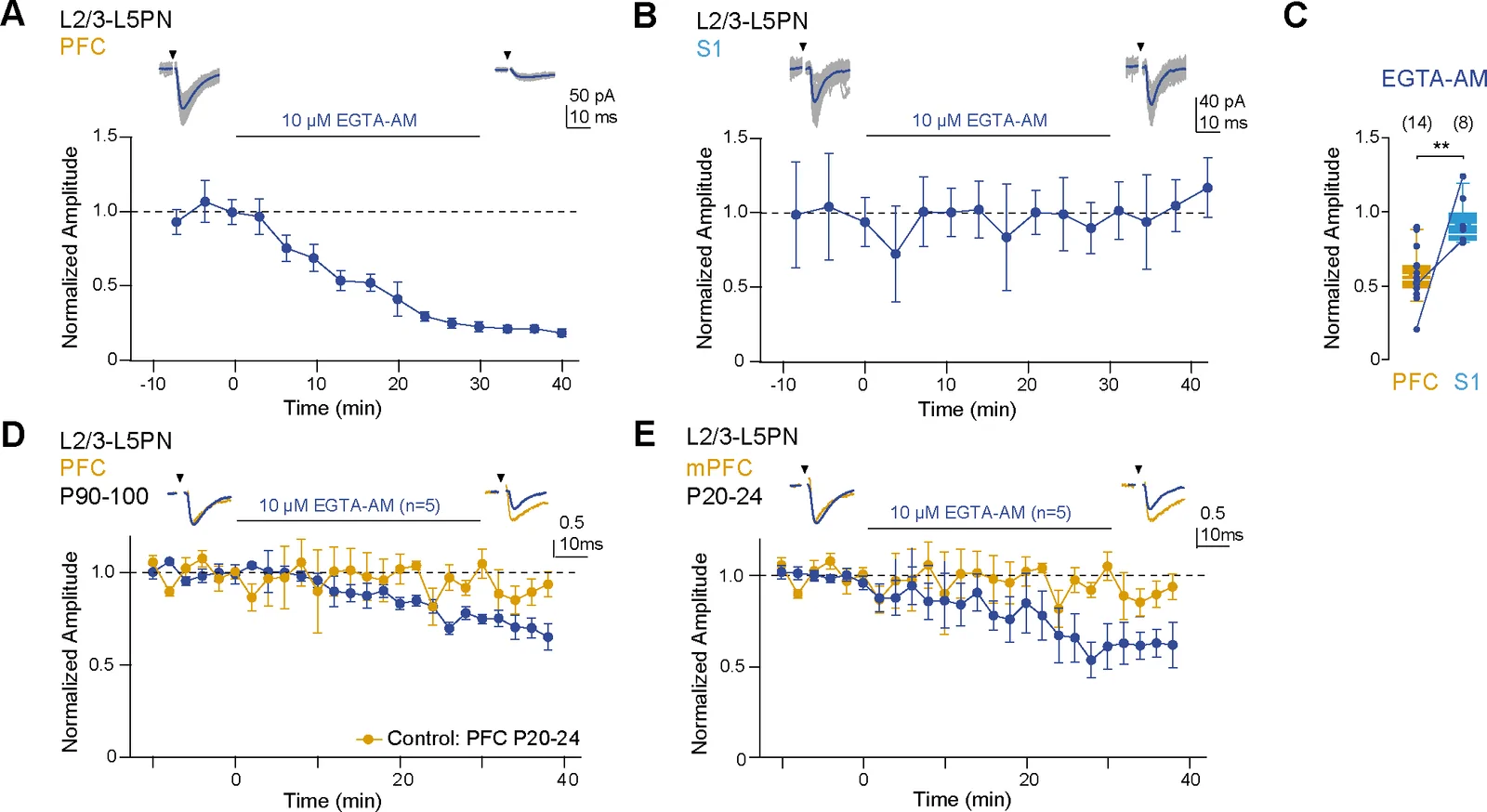
EGTA sensitivity in prefrontal cortex (PFC). (**A, B**) Example recording of baseline-normalized EPSC amplitudes (means ± SDs, 20 EPSCs each were binned) recorded from an L2/3-layer 5 pyramidal neuron (L5PN) connection in PFC (**A**) and primary somatosensory cortex (S1) cortex (**B**) derived from the same mouse (P24). Following baseline recordings (10 min), slices were perfused for 30 min (solid line) with artificial cerebrospinal fluid (ACSF) containing 10 μM EGTA-AM (blue), and thereafter rinsed for 10 min with ACSF (test period). Insets: EPSCs (averages in blue, individual recordings in gray) during baseline and test period. (**C**) Boxplot summarizing EGTA-AM effects in PFC and S1. Individual experiments are included as data points; data obtained from the same mouse were connected by a line. Note the significant EGTA sensitivity in PFC (**p=0.002, Mann-Whitney U test [MWU]). (**D**) Averaged, baseline-normalized EPSCs (2 min bins) recorded from L2/3-L5PN connections in PFC of 90- to 100-day-old mice as in Figure 2C. Insets: Averaged EPSCs during baseline and test period. (**E**) Same as in (**D**), but for L2/3-L5PN connections in medial PFC (mPFC) of 21- to 26-day-old mice .

We proceeded by comparing the EGTA sensitivity of release at L2/3-L5PN synapses between S1 and PFC, using extracellular stimulation and incubation with Ca^2+^ chelator-AM compounds (Figure 2C–F; Baur et al., 2015; Kusch et al., 2018). Following 10 min of stable baseline recordings, slices were perfused with artificial cerebrospinal fluid (ACSF) that either contained the solvent DMSO/Pluronic alone (control) or 10 µM of dissolved EGTA-AM or BAPTA-AM. The fast BAPTA interferes with release irrespective of the coupling topography, thereby providing a positive control for the proper functioning of the AM-method (Eggermann et al., 2012). EPSC amplitudes were quantified during a subsequent 10 min period with perfusion with normal ACSF. During this test period, BAPTA significantly reduced EPSC amplitudes both in PFC (0.28, 0.12–0.32; control: 0.99, 0.84–1.02) and in S1 (0.08, 0–0.18; control: 0.85, 0.83–0.92). On the other hand, EGTA significantly reduced EPSC amplitudes only in PFC (0.54, 0.47–0.67; 55% of control) but not in S1 (0.85, 0.81–1.04; 100% of control). For a better comparison of EGTA effects, some recordings were performed in PFC and S1 derived from the same animal to rule out interindividual effects (see example recording in Figure 2—figure supplement 1A–C).

In S1, we previously found that coupling switches from loose to tight during postnatal development between ~P10 and ~P20 (Bornschein et al., 2019a). To probe for a developmental retardation of this process in PFC, we extended the EGTA-AM experiments at L2/3-L5PN synapses to the older age window of P90–100. Also, in these experiments, we found a significant sensitivity of release to EGTA (0.76, 0.65–0.78; Figure 2D, Figure 2—figure supplement 1D), indicating that loose coupling is a functionally persistent feature of synapses in PFC. Finally, we also extended the EGTA-AM experiments to synapses in mPFC and again found significant effects of EGTA on the EPSC amplitudes (0.65, 0.49–0.75; Figure 2D, Figure 2—figure supplement 1E).

Together, the results obtained with the slow Ca^2+^ chelator EGTA suggest that major glutamatergic synapses in mature PFC operate with loose coupling in contrast to tight coupling in the mature S1. The data further suggest that loose coupling is a developmentally persistent property of synapses in PFC. Finally, they indicate that the above functional differences between synapses in S1 and PFC originate from the differences in Ca^2+^ influx-release coupling.

### Release probabilities are similar in PFC and S1

**Figure 3.**
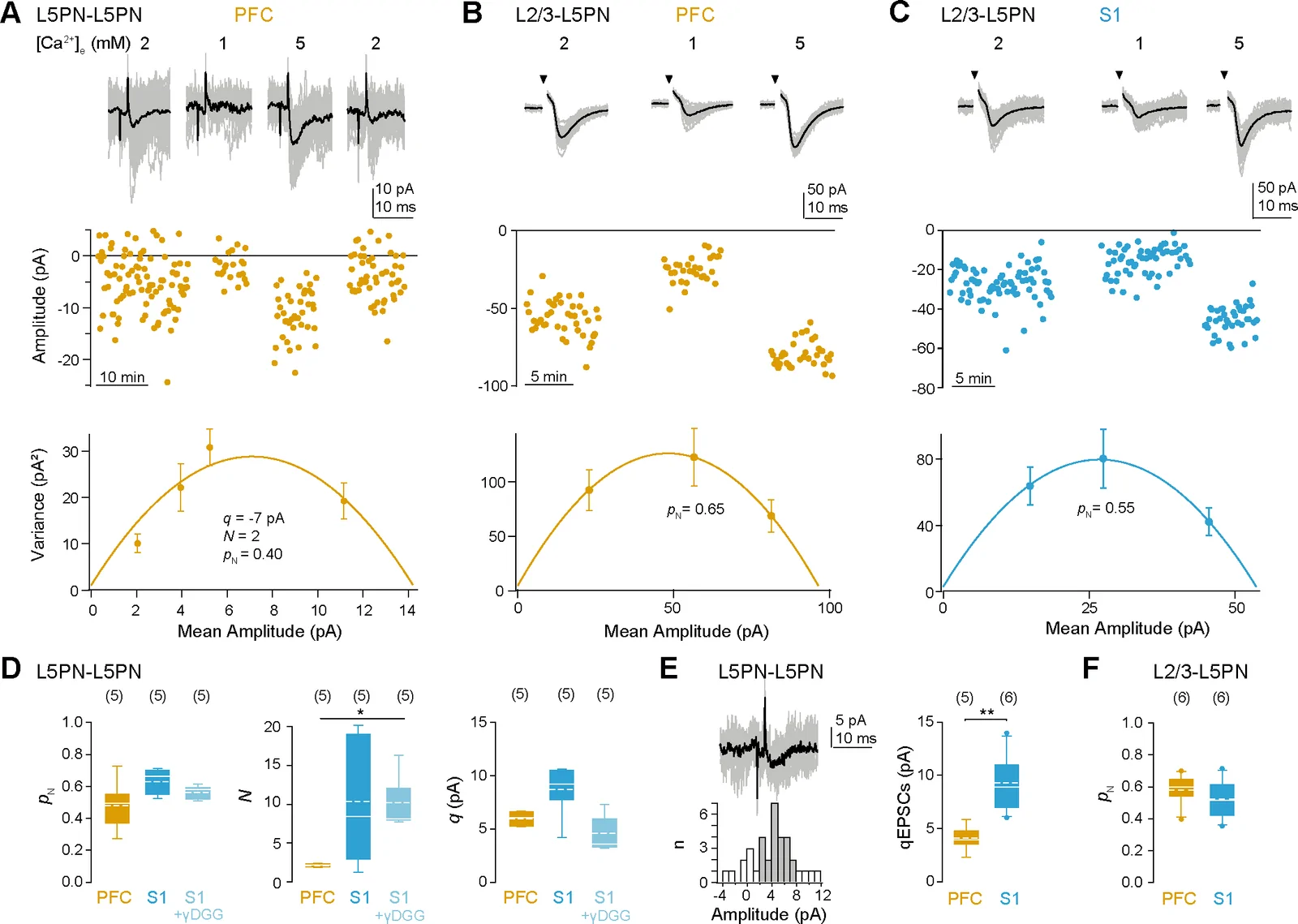
Quantal synaptic parameters in prefrontal cortex (PFC) and primary somatosensory cortex (S1). (**A**) Multiple probability fluctuation analysis (MPFA) of EPSC amplitudes recorded at the indicated [Ca^2+^]_e_ from a pair of layer 5 pyramidal neurons (L5PNs) in PFC. Top: EPSCs (gray, average in black) recorded in 1, 2, and 5 mM [Ca^2+^]_e_. Middle: Plot of EPSC amplitudes over time. Bottom: Corresponding mean-variance plot fitted with a parabola estimating the quantal parameters of release. *p*_N_ is for 2 mM [Ca^2+^]_e_. (**B**) As in (**A**), but for an L2/3-L5PN connection in PFC. Recordings were made in the presence of 0.25 mM kynurenic acid (Kyn) and 50 µM 2-amino-5-phosphonovaleric acid (APV). (**C**) As in (**B**), but in S1. (**D**) Summary of quantal release parameters in L5PN pairs from PFC and S1 and in S1 with 1–2 mM γ-DGG (γ-D-glutamylglycine) (S1 data from Bornschein et al., 2019a; p=0.121, *p=0.048, p=0.054, ANOVA on ranks). (**E**) Left: Example of evoked qEPSCs (top) and the corresponding amplitude histogram (bottom, gray range was used for calculating the qEPSC amplitude). Right: Summary of qEPSC amplitudes in PFC and S1 (**p=0.004, Mann-Whitney U test [MWU]). (**F**) Summary of *p*_N_ values in L2/3-L5PN connections from PFC and S1 (p=0.485, MWU). Figure 3—source data 1.Data from recordings of L5PN-L5PN connections corresponding to Figure 3A, D and E. Figure 3—source data 2.Data from recordings of L2/3-L5PN connections corresponding to Figure 3B, C and F.

To test whether the release probabilities (*p*_N_) of the differentially coupled release sites in PFC and S1 differ, we performed multiple probability fluctuation analysis (MPFA) (Clements and Silver, 2000; Brachtendorf et al., 2025) at different extracellular Ca^2+^ concentrations ([Ca^2+^]_e_; Figure 3; Bornschein et al., 2019a). The parabolic MPFA-fits to variance-mean plots from recordings of pairs of L5PNs in PFC yielded a median *p*_N_ of 0.50 (0.37–0.55; Figure 3A and D). This value was slightly but not significantly smaller than *p*_N_ of 0.66 (0.55–0.70), which we quantified previously for this connection in S1 (Bornschein et al., 2019a).

We also quantified *p*_N_ at L2/3-L5PN synapses, both in PFC and in S1, using MPFA with extracellular stimulation (Baur et al., 2015). We found that *p*_N_ was as high as at the L5PN-L5PN synapses in both areas with values (PFC: 0.60, 0.51–0.66; S1: 0.52, 0.40–0.64) being not significantly different between areas (Figure 3B, C, and F). Interestingly, in our previous study (Bornschein et al., 2019a), we found that the developmental switch from loose to tight coupling at L5PN synapses in S1 also had no significant impact on *p*_N_.

From MPFA in paired recordings, it is possible to estimate the number of release sites (*N*) and the quantal size (*q*), additionally. *N* was significantly smaller in PFC than in S1 being only 2.1 (1.9–2.3) rather than 8 (3–19). *q* tended to be smaller in PFC (6 pA, 5–7 pA) than in S1 (9 pA; 8–11 pA), and the difference became significant when evoked quantal EPSCs recorded in low [Ca^2+^]_e_ were compared (PFC: 4 pA, 4–5 pA; S1: 9 pA, 7–11 pA). We consider it unlikely that postsynaptic receptor saturation or desensitization influenced the determination of quantal parameters since experiments with the competitive low-affinity glutamate receptor antagonist γ-DGG (γ-D-glutamylglycine) (Chanda and Xu-Friedman, 2010) performed at the S1 synapses had revealed no signs of this (*p*_N_ = 0.58, 0.52–0.60; *N*=8, 8–12; Figure 3D). Due to the smaller size of the EPSCs, testing on the PFC synapses was not possible. Estimating EPSC amplitudes (EPSC = *N p*_N_ *q*; PFC, 6 pA; S1, 48 pA) and failure rates (*F* = (1*–p*_N_)^*N*; PFC, 0.25, S1, 0.0001) from the quantal parameters yielded values similar to those recorded (Figure 1). The determined parameters were therefore well suited to explain the area-specific differences in synaptic efficacy during individual action potentials.

### Presynaptic Ca^2+^ signals are similar in PFC and S1

**Figure 4.**
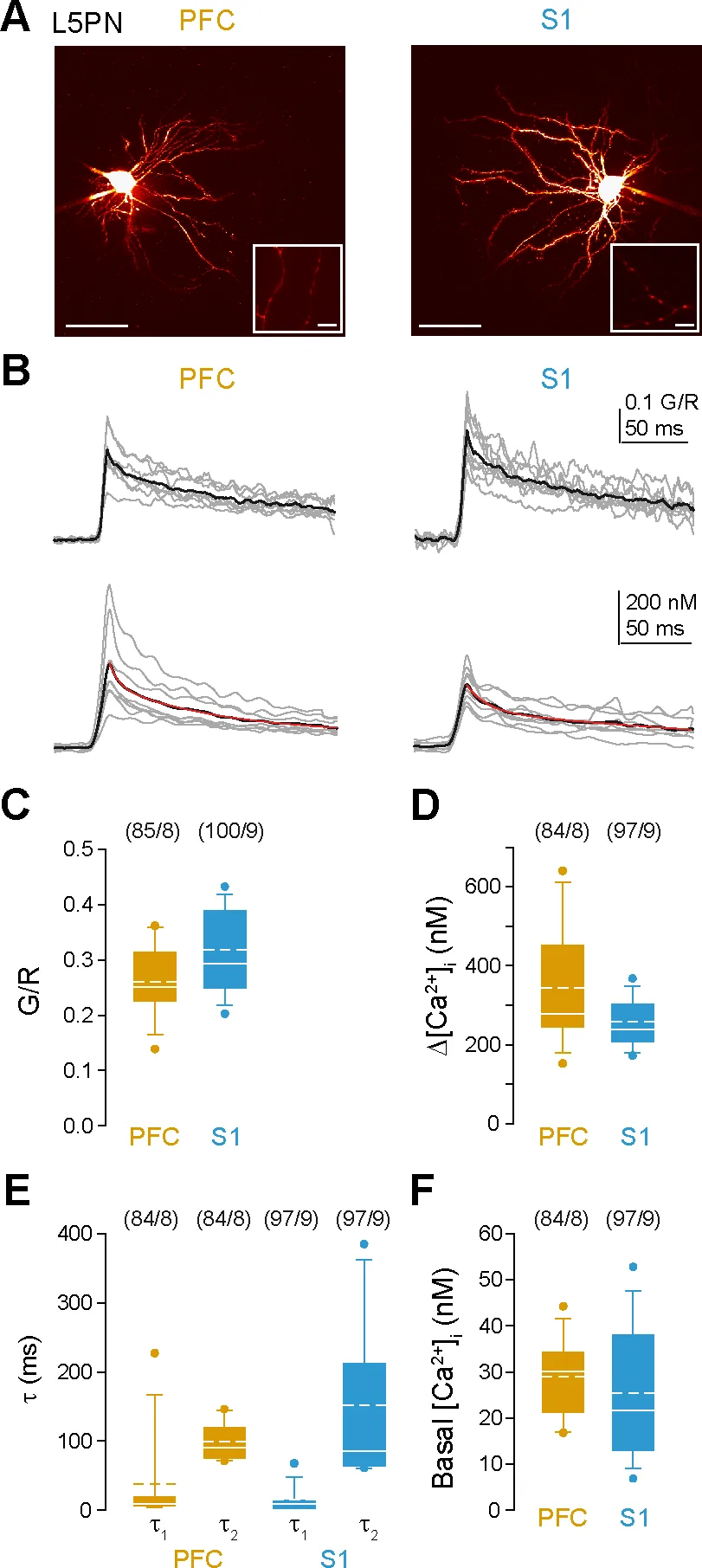
Presynaptic Ca^2+^ transients are similar in prefrontal cortex (PFC) and primary somatosensory cortex (S1). (**A**) Two-photon images of layer 5 pyramidal neurons (L5PNs) in PFC (left) and S1 (right) filled with Fluo-5F and Alexa 594 (scale bar 50 µm). Insets: Magnifications showing presynaptic boutons from which Ca^2+^ transients are recorded (scale bar 5 µm). (**B**) *G*/*R* signals (top) evoked by single APs in individual cells (gray, average of 6–15 boutons each, grand average in black) and their conversion to Ca^2+^ increases (∆[Ca^2+^]_i_, bottom). (**C–F**) Summary of *G*/*R* signals (**C**; p=0.112, Mann-Whitney U test [MWU]; numbers of boutons and cells in brackets), ∆[Ca^2+^]_i_ (**D**; p=0.361), decay time constants *τ*_1_ and *τ*_2_ (**E**; p=0.532, 0.962), and basal ∆[Ca^2+^]_i_ (**F**; p=0.597) in PFC and S1. Note that there is no significant difference in presynaptic Ca^2+^ transients between PFC and S1. Figure 4—source data 1.Data from calcium imaging experiments in L5PN boutons corresponding to Figure 4C-F.

For a thorough interpretation of the above results, knowledge about the presynaptic Ca^2+^ dynamics in the different synapses is required. We performed dual-dye two-photon Ca^2+^ imaging (Sabatini et al., 2002) to quantify volume-averaged Ca^2+^ signals at presumed presynaptic boutons located on axon collaterals of L5PNs in PFC and in S1. The imaged presynaptic boutons most likely connect to neighboring pyramidal cells, as connectivity between L5PNs in layer 5A is high (Markram et al., 1997; Frick et al., 2008; Feldmeyer, 2012). Nevertheless, a proportion of interneurons as postsynaptic targets cannot be ruled out (Beaulieu, 1993; Meyer et al., 2011). We quantified single action potential-mediated elevations in green over red fluorescence signals (Δ*G*/*R*) that were converted to increases in intracellular calcium (∆[Ca^2+^]_i_) (Figure 4; Bornschein et al., 2019a). We found that neither the Ca^2+^ transients nor the decay time constants were significantly different between the cortical areas (Figure 4A–E; PFC: *G*/*R*=0.25, 0.23–0.31; ∆[Ca^2+^]_i_=279 nM, 244–453 nM; *τ*_decay,1_=9 ms, 6–19 ms; *τ*_decay,2_=91 ms, 75–120 ms; S1: *G*/*R*=0.29, 0.25–0.39; ∆[Ca^2+^]_i_=238 nM, 208–303 nM; *τ*_decay,1_=9 ms, 3–15 ms; *τ*_decay,2_=86 ms, 63–212 ms). The quantification of absolute basal [Ca^2+^]_i_ was limited by the *K*_D_ of Fluo5F (439 nM), which slightly underestimated basal [Ca^2+^]_i_ values in comparison to quantification with OGB1 (*K*_*D*_ = 166 nM, Figure 4—figure supplement 1). Nevertheless, relative comparison of basal [Ca^2+^]_i_ yielded no significant differences between PFC (30 nM, 21–34 nM) and S1 (22 nM, 13–38 nM; Figure 4F).

**Figure 4—figure supplement 1.**
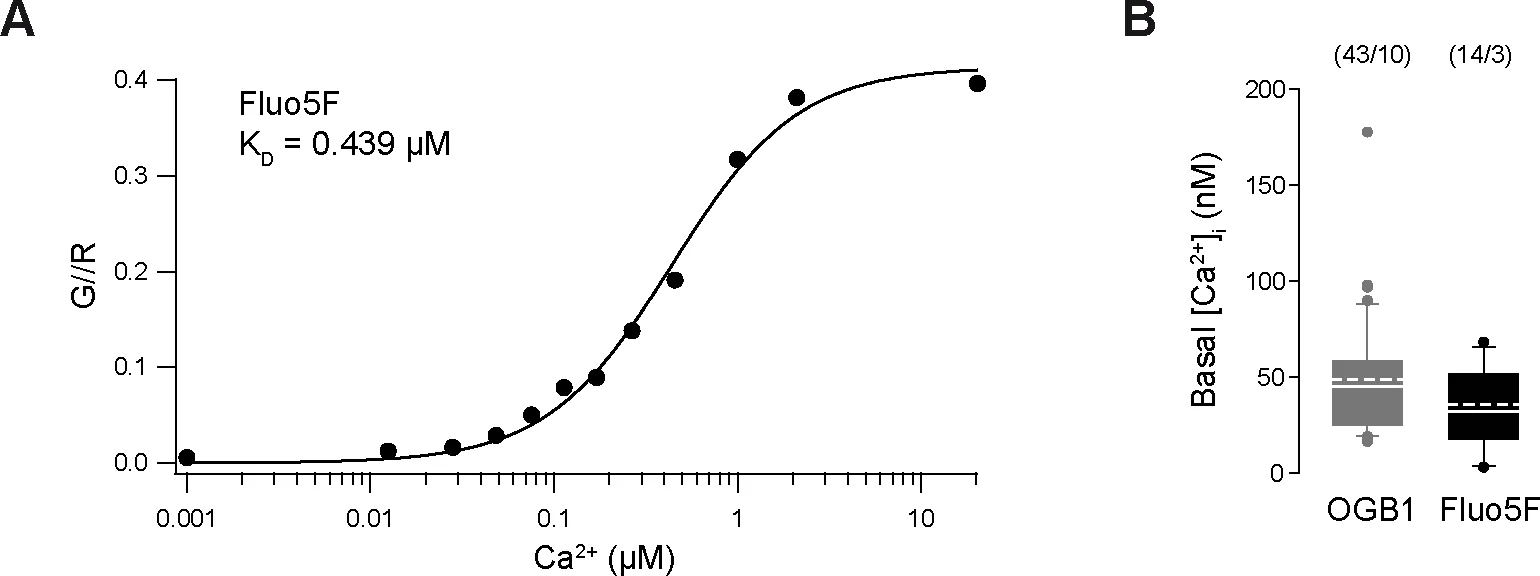
In vitro calibration curve and basal ∆[Ca^2+^]_i_. (**A**) Calibration curve of Fluo-5F in a K-gluconate-based pipette solution. The *K*_D_ value of 439 nM was derived from a Hill fit (solid curve). (**B**) Comparison of basal [Ca^2+^]_i_ determined with Fluo-5F and OGB-1, respectively (p=0.204, Mann-Whitney U test [MWU]). Figure 4—figure supplement 1—source data 1.Data from calcium imaging experiments in L5PN boutons corresponding to Figure 4—figure supplement 1A. Figure 4—figure supplement 1—source data 2.Data from calcium imaging experiments in L5PN boutons corresponding to Figure 4—figure supplement 1B.

The similarity in the presynaptic Ca^2+^ transients of boutons in PFC and S1 suggests that differences in the presynaptic Ca^2+^ influx are unlikely to be the cause for the differential Ca^2+^ chelator effects. Rather, they support the view that these differences result from loose coupling in PFC and from tight coupling in S1.

### Estimate of the coupling distance in PFC verify loose microdomain coupling in PFC

**Figure 5.**
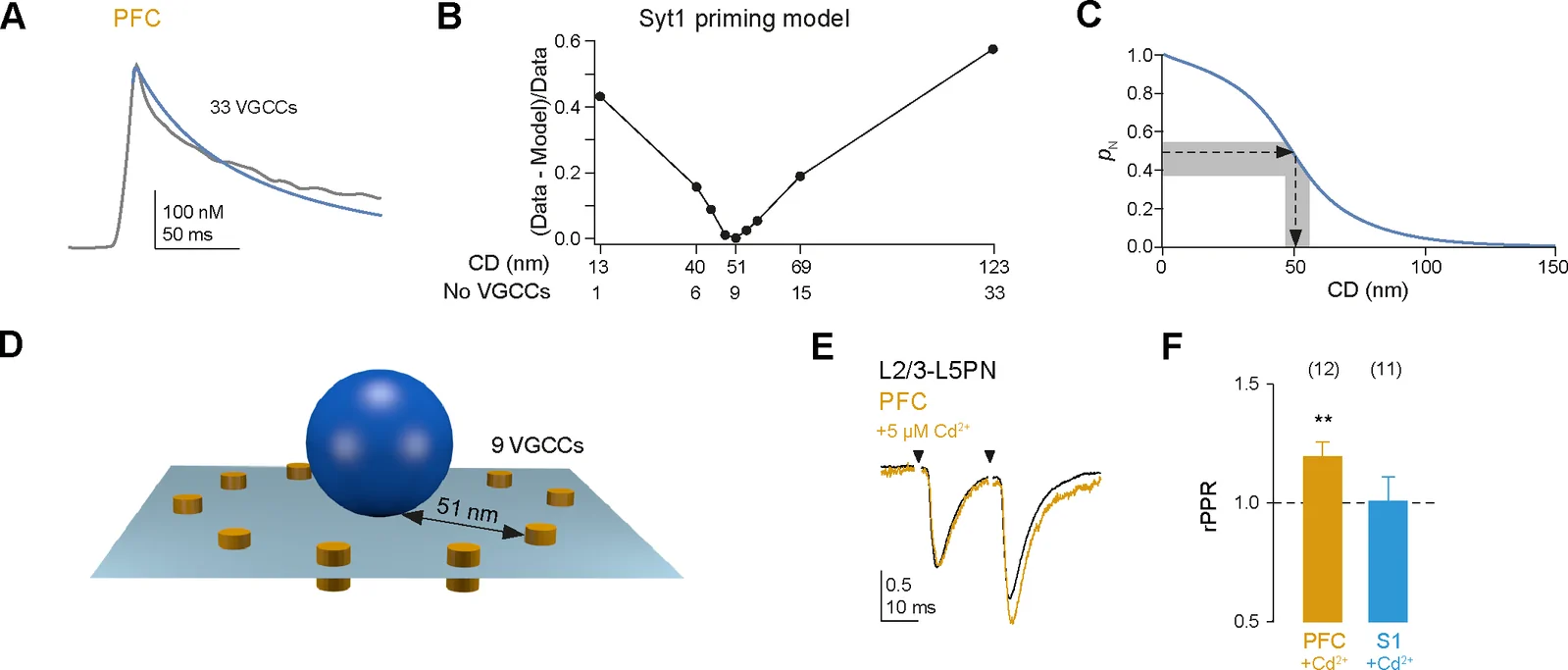
Model predicts microdomain coupling at prefrontal cortex (PFC) synapses. (**A**) Fit of the model (blue line) to the measured average Ca^2+^ signal (**A**; gray line) of layer 5 pyramidal neuron (L5PN) boutons in PFC. The fit was obtained with the indicated number of open voltage-gated Ca^2+^ channels (VGCCs). (**B**) *p*_N_ values were simulated with the Syt1 priming model (Bornschein et al., 2019a; Bornschein et al., 2025) for control conditions and in the presence of EGTA with different VGCC numbers yielded different coupling distances (CDs) in the model. The simulated *p*_N_ were subtracted from and normalized to the experimental values, i.e., a value close to zero indicates best agreement between simulation and experiment. This was obtained with nine open VGCCs at a CD of 51 nm (see **C**). (**C**) Simulated *p*_N_ for nine open VGCCs under control conditions at increasing CDs (blue line). The experimental *p*_N_ value (median ± IQR, dashed line, gray range, Figure 3D) was reproduced at a CD of 51 nm (48–55 nm). (**D**) Illustration of the ring model used in **B** and **C** with a CD of 51 nm between nine VGCCs (orange) and the release sensor of a vesicle (blue). (**E**) Examples of averaged normalized paired pulse recordings at 50 Hz at L2/3-L5PN synapses before (black) and after (orange) application of 5 µM Cd^2+^ in PFC. (**F**) Bar graph quantifying normalized paired pulse ratios (PPRs) after Cd^2+^ application in PFC and primary somatosensory cortex (S1) (**p=0.005, p=0.667, paired t-test; Shapiro-Wilk normality test: p=0.514). Note that Cd^2+^ increased the PPR in PFC. Figure 5—source data 1.Data from recordings of L2/3-L5PN connections corresponding to Figure 5F.

To derive a quantitative picture of the coupling topography in L5PN synapses in PFC, we used numerical computer simulations that were based on and constrained by the experimental data. First, we simulated the measured Ca^2+^ transient with the indicator dye present in the simulation (Figure 5A). Subsequently, the indicator dye was removed, and a release sensor model for Synaptotagmin-1-triggered release from L5PN boutons was included in the simulations (Figure 5B and C, Figure 5—figure supplement 1; Bornschein et al., 2025). VGCCs were assumed to form a ring-like structure around a vesicle (Figure 5D), similar to the release site topography in young S1 (Bornschein et al., 2019a). Release rates as reported by the sensor model were integrated over time to yield the *p*_N_ values. The coupling distance between the sensor and the ring of VGCCs, as well as the number of VGCCs forming the ring, were iteratively varied until the simulation correctly predicted the experimental *p*_N_ values from MPFA obtained under control conditions and in the presence of EGTA. By this procedure, we quantified an average coupling distance of 48–51 nm between a microdomain of several VGCCs and the release sensor (Figure 5B, Figure 5—figure supplement 1), similar to the estimate for these synapses in immature S1 (Bornschein et al., 2019a). Thus, a microdomain model similar to immature S1 is suitable to predict the data from mature PFC, whereas the same synapses in mature S1 operated with Ca^2+^ signaling nanodomains in which only one to three VGCCs at distances of 11–16 nm trigger fusion (Bornschein et al., 2019a; Bornschein et al., 2025).

**Figure 5—figure supplement 1.**
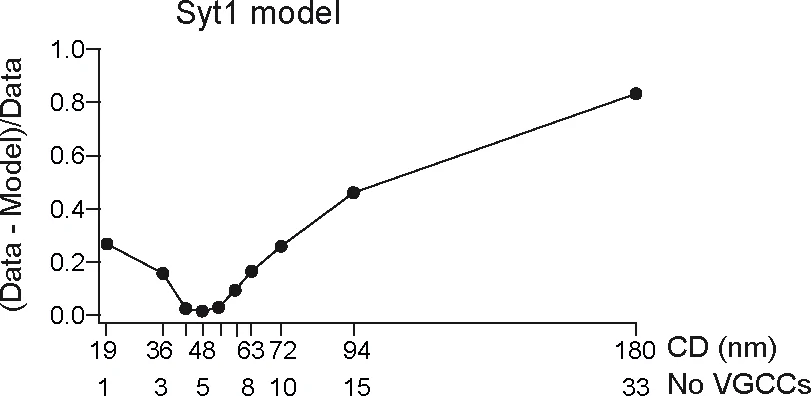
Prediction of the coupling distance with an alternative Syt1 model. As in Figure 5B but instead of the Syt1 priming model, the Syt1 model (Bornschein et al., 2019b; Bornschein et al., 2025) was used for simulating the release rates and the corresponding *p*_N_ values.

To experimentally test whether overlapping Ca^2+^ domains from several VGCCs trigger release in PFC (microdomain), we analyzed the effects of the unspecific VGCC blocker Cd^2+^ onto the PPR. If fusion is triggered by such VGCC microdomains, application of a subsaturating concentration of Cd^2+^ will increase the PPR, while it will leave PPR unaffected if only a single or few VGCCs trigger release (Hefft et al., 2002; Bucurenciu et al., 2008; Scimemi and Diamond, 2012; Baur et al., 2015; Bornschein et al., 2019a). In paired pulse experiments at L2/3-L5PN synapses in PFC, we indeed found a significant increase in the PPR following the application of 5 µM Cd^2+^ (rPPR = 1.20 ± 0.06), whereas the average PPR remained unaffected in S1 (rPPR = 1.01 ± 0.10; Figure 5E and F). Thus, our data and simulations suggest that the nanotopography of PN synapses in mature PFC is reminiscent of the same synapses in young S1.

## Discussion

Our results provide evidence that the same archetypes of glutamatergic synapses show area-specific differences in their functional release site nanoarchitectures in the mature neocortex. They suggest loose microdomain coupling as a synaptic correlate of higher-order neocor´ical functions in PNs.

Within a given layer, we did not make any further distinctions between PN subtypes, either morphologically or based on firing patterns. However, within a cortical area (S1 or PFC), we did not find a difference between glutamatergic synapses from L2/3 onto L5PNs and L5PN to L5PN synapses, neither with regard to the EGTA sensitivity of release nor with regard to *p*_N_. In particular, we found homogeneous results and similar variability in both, the examined connections in the PFC and in S1, with no discernible clustering in the data that would indicate stimulation of different cell populations. These findings suggest that excitatory inputs to L5PNs exhibit similar properties (PPR, *p*_N_, CD) irrespective of whether they originate in L2/3 or in neighboring PNs in L5A. However, we do see significant differences between synapses in the different cortical areas S1 and PFC. Thus, intra-area specific differences in morphology and spiking patterns among PNs appear to be not reflected on the level of their synapses.

The differences in coupling gave rise to larger variability of synaptic delays (SD_Delay_) in PFC compared to S1 and were associated with differences in synaptic reliability and efficacy, whereas the *p*_N_ values were not significantly different between areas. A major determinant of *p*_N_ is the size of the Ca^2+^ signal at the release sensor. Accordingly, tightening of coupling can increase *p*_N_ (Eggermann et al., 2012; Baur et al., 2015; Bornschein and Schmidt, 2019c). However, large Ca^2+^ elevations at the release sensor can also be obtained with Ca^2+^ microdomains. At L5PN-L5PN synapses in S1, the developmental switch from microdomain to nanodomain coupling had no effect on *p*_N_ (Bornschein et al., 2019a). At the calyx of Held (Iwasaki and Takahashi, 2001; Taschenberger et al., 2002; Fedchyshyn and Wang, 2005; Koike-Tani et al., 2008; Nakamura et al., 2015) and at cerebellar basket cell to Purkinje cell synapses (Chen et al., 2024), *p*_N_ even decreased during development, whereas coupling got tightened. The decrease in *p*_N_ was compensated by an increase in *N* at these synapses, which increased their reliability and efficacy during development. We found *N* to be larger in S1 than in PFC. Thus, it appears that differences in coupling are typically associated with other characteristic changes in the release parameters. Overall, this suggests that reliable CNS synapses switch from an initial state with high plasticity to a matured state with high reliability. On the other hand, PN synapses in the PFC appear to remain in a more juvenile state that favors plasticity over reliability.

Nanodomain coupling was also found in the peripheral nervous system, in particular at retinal (Singer and Diamond, 2003; Jarsky et al., 2010) and auditory (Moser and Beutner, 2000; Brandt et al., 2005) ribbon type synapses and at the neuromuscular junction (Harlow et al., 2001; Shahrezaei et al., 2006). These synapses have highly specialized properties and appear to be optimized for very reliable transmission and, in the case of ribbon synapses, also for high-frequency coding of sensory information (reviewed in Matthews and Fuchs, 2010; Eggermann et al., 2012). Thus, it appears that synapses in the sensory pathways, in particular those engaged in reliable high-frequency coding of sensory information, both in the periphery and in the lower processing stages of the CNS, up to primary sensory cortices, operate with nanodomain coupling. In the executing motor pathway, the neuromuscular junction uses nanodomain coupling and, as recent results from our group suggest, also PNs in the primary motor cortex (Yarim et al., 2026). It is tempting to speculate that complete loops from or to the primary cortices to their peripheral target organs operate with nanodomains. Microdomain coupling, on the other hand, appears to come into play only if integration of information from multiple sources and plasticity are the main focus, as at certain synapses in PFC (this study) or hippocampus (Vyleta and Jonas, 2014).

The microdomain was assumed to be formed by a ring-like structure of VGCCs around a vesicle (Figure 5D). This topography was chosen because such a microdomain was found to best predict the experimental data of transmitter release from PNs in young S1 (Bornschein et al., 2019a). Other previously described distributions of VGCCs suitable to reproduce release data cover random distributions of VGCCs (Scimemi and Diamond, 2012), VGCC clusters (Meinrenken et al., 2002; Nakamura et al., 2015; Rebola et al., 2019), and exclusion zones (Keller et al., 2015; Rebola et al., 2019). In the early S1, all of these models predicted a higher EGTA sensitivity of the microdomain; however, these models provided a poorer fit to the full set of the experimental data than the ring-like structure (Bornschein et al., 2019a). Since the experimental data from PNs in the mature PFC were similar to those in young S1, these other microdomain models were not tested explicitly here.

Differences in the sensitivity of release to low to moderate concentrations of EGTA (≤30 mM) are a standard indicator of differences in the coupling distance (e.g. Adler et al., 1991; Bucurenciu et al., 2008; reviewed in Eggermann et al., 2012; Vyleta and Jonas, 2014; Kusch et al., 2018; Bornschein et al., 2019a). *p*_N_ is determined by the size of the Ca^2+^ signal at the release sensor and the binding kinetics and affinity of the sensor. The former in turn is determined by the details of the Ca^2+^ influx and the diffusional coupling distance between the VGCCs and the sensor. The similarity of Ca^2+^ signals between synapses in PFC and S1 (Figure 4) indicates that Ca^2+^ influx is similar between boutons, although more subtle differences in the influx kinetics may have remained undetected in these volume-averaged signals. Regarding sensor affinity, results in a previous study indicate that differences in EGTA sensitivity show differences in coupling rather than sensor affinity even if *k*_on_ of the sensor and its affinity should differ as much as tenfold, which appears to be an unlikely scenario given that even the two major isoforms of Synaptotagmin that trigger synchronous release differ by less than a factor of three to four in their affinity (Bollmann et al., 2000; Schneggenburger and Neher, 2000; Bornschein et al., 2025). Finally, the increase in the PPR induced by the application of Cd^2+^ further supports our conclusion of microdomain coupling in the PFC synapses (Scimemi and Diamond, 2012). Thus, although the absolute EGTA sensitivity is influenced by different factors, which necessitates data-constrained models for quantitative comparisons, the general sensitivity of release to EGTA indicates loose coupling.

The differences we observed in short-term plasticity suggest that S1 emphasized the first transmission process, whereas in PFC facilitation emphasized the subsequent release. The mechanisms of short-term plasticity are complex and include the regulation of *p*_N_, the speed of vesicle replenishment, and the recruitment of release sites (Neher and Brose, 2018; Schmidt, 2019; Neher, 2024; Brachtendorf et al., 2025). Classically, *p*_N_ was considered as the major determinant of short-term plasticity (e.g. reviewed in Zucker and Regehr, 2002; Feldmeyer and Radnikow, 2009). More recently, other factors, including the number of occupied release sites, their replenishment, or an increase in their occupancy, or the expression of endogenous Ca^2+^ buffers have been considered as more important determinants of short-term plasticity (Rozov et al., 2001; Blatow et al., 2003; Felmy et al., 2003; Matveev et al., 2004; Bornschein et al., 2013; Miki et al., 2016; Doussau et al., 2017; Jackman and Regehr, 2017; Neher and Brose, 2018). Irrespective of the detailed mechanism, we consider the differential emphasis on the available presynaptic efficacy between PFC and S1 as a signature of higher plasticity and flexibility in the processing of information in PFC compared to S1.

Short-term plasticity changes during postnatal development at different cortical PN connections without alterations in *p*_N_ (Reyes and Sakmann, 1999; Bornschein et al., 2019b). For L5PN to L5PN connections, these differences were found to result from the maturation of an intermediate replenishment vesicle pool (Bornschein et al., 2019b). Such pool maturation may also underlie the elimination of layer-specific differences in short-term plasticity between L2/3-L5B and L5B-L5B synaptic connections that were evident in young rats but eliminated during the first weeks of postnatal development (Reyes and Sakmann, 1999). Since postnatal development follows the same time course in mouse PFC and S1 and occurs predominantly within the first 2 weeks after birth, significant layer-specific differences in PN synapses are unlikely in both areas in our experimental time window (Kroon et al., 2019). Consistently, we found similar PPRs at L2/3-L5PN synapses and L5PN-L5PN synapses in both areas, with facilitation in PFC and depression in S1, irrespective of the presynaptic PN synapse type.

Two other previous studies on L5APN (Frick et al., 2008) and L5BPN (Markram et al., 1997) connections concluded that these synapses operate with high release probability, which nicely agrees with our previous (Bornschein et al., 2019a) and current results. It is remarkable that we did not even detect any differences between the L2/3-L5PN and L5PN-L5PN synapses within a given cortical area. Overall, these results from different studies (Markram et al., 1997; Reyes and Sakmann, 1999; Frick et al., 2008; Bornschein et al., 2019b; Bornschein et al., 2019a) may indicate that variability on the synaptic level between PNs of a given area is not pronounced. In order to further substantiate this, we determined the relative variability in median EPSC amplitudes to test whether there is a higher variability of recorded cell types in PFC compared to S1. The relative MAD (median absolute deviation) of EPSC amplitudes was 0.46 in PFC and 0.50 in S1. The similarity in these values argues against higher cell-type variability in PFC compared to S1.

Our results have implications for long-term plasticity at neocortical PN synapses. Both long-term potentiation (LTP) and depression (LTD) have a presynaptic locus at these synapses and likely involve changes in *p*_N_, but the final steps that alter release are not well understood (Feldman, 2009; Castillo, 2012; Feldman, 2012). A recent study on L5PN synapses in S1 showed an increase in the number of primed vesicles as a major mechanism of LTP (Weichard et al., 2023). PN synapses in S1 are already tightly coupled and therefore a further decrease in the coupling distance is an unlikely mechanism of LTP. However, this does not exclude that in S1 LTD induction breaks tight coupling and vice versa that LTP in PFC involves tightening of coupling. In addition, in PFC we found an increased contribution of NMDAR on the postsynaptic site. Thus, these synapses have increased potential for plasticity on the pre- and the postsynaptic site. During induction of long-term plasticity, the postsynaptic site signals back to the presynaptic site via retrograde messengers (Regehr et al., 2009), and loose coupling will provide the substrate for enhanced regulatory capacity (Vyleta and Jonas, 2014).

In general, loose coupling provides more flexibility to regulate [Ca^2+^]_i_ at the release sensor (Vyleta and Jonas, 2014). Prefrontal networks carry out higher-order computations that transform sensory information and memory to perform flexible output such as delayed response, inference, and planning (Narayanan et al., 2025). Our findings suggest that loose coupling provides a synaptic foundation for such flexible neocortical network functions.

## Methods

**Key resources table:** 

Reagent type (species) or resource	Designation	Source or reference	Identifiers	Additional information
Strain, strain background (*Mus musculus*, female/male)	C57BL/6J	Animal Facility, Medical Faculty, University of Leipzig	N/A	P21–26 and P90–100
Chemical compound, drug	NaCl	Merck	Cat. # 1.06400.1000, RRID:SCR_001287	
Chemical compound, drug	KCl	Sigma-Aldrich	Cat. # P9333, RRID:SCR_008988	
Chemical compound, drug	NaH_2_PO_4_	Merck	Cat. # 1.06349.1000, RRID:SCR_001287	
Chemical compound, drug	NaHCO_3_	Roth	Cat. # 6885.1, RRID:SCR_005711	
Chemical compound, drug	MgCl_2_	Sigma-Aldrich	Cat. # M2670, RRID:SCR_008988	
Chemical compound, drug	CaCl_2_	Sigma-Aldrich	Cat. # C5080, RRID:SCR_008988	
Chemical compound, drug	Glucose	Roth	Cat. # 6780.1, RRID:SCR_005711	
Chemical compound, drug	(-)-Bicuculline methiodide	Tocris	Cat. # 2503, RRID:SCR_003689	
Chemical compound, drug	K-gluconate	Sigma-Aldrich	Cat. # G4500, RRID:SCR_008988	
Chemical compound, drug	Na_2_ATP	Sigma-Aldrich	Cat. # A26209, RRID:SCR_008988	
Chemical compound, drug	NaGTP	Sigma-Aldrich	Cat. # G8877, RRID:SCR_008988	
Chemical compound, drug	EGTA	Sigma-Aldrich	Cat. # E0396, RRID:SCR_008988	
Chemical compound, drug	KHEPES	Sigma-Aldrich	Cat. # H0527, RRID:SCR_008988	
Chemical compound, drug	KOH	Roth	Cat. # K017.1, RRID:SCR_005711	
Chemical compound, drug	HCl	Roth	Cat. # K024.1, RRID:SCR_005711	
Chemical compound, drug	DMSO	Sigma-Aldrich	Cat. # D8418, RRID:SCR_008988	
Chemical compound, drug	Pluronic F-127	Molecular Probes	Cat. # P3000MP, RRID:SCR_013318	
Chemical compound, drug	EGTA-AM	Invitrogen, Thermo Fisher	Cat. # E1219, RRID:SCR_008452	
Chemical compound, drug	BAPTA-AM	Invitrogen, Thermo Fisher	Cat. # B6769, RRID:SCR_008452	
Chemical compound, drug	CdCl_2_	Sigma-Aldrich	Cat. # 655198, RRID:SCR_008988	
Chemical compound, drug	2-Amino-5-phosphonovaleric acid	Alomone labs	Cat. # D-145, RRID:SCR_013570	
Chemical compound, drug	Kynurenic acid	Sigma-Aldrich	Cat. # K3375, RRID:SCR_008988	
Chemical compound, drug	Fluo-5F	Invitrogen, Thermo Fisher	Cat. # F14221, RRID:SCR_008452	
Chemical compound, drug	OGB-1	Invitrogen, Thermo Fisher	Cat. # O6806, RRID:SCR_008452	
Chemical compound, drug	Atto-594	Atto-Tec, Sigma-Aldrich	Cat. # 05676, RRID:SCR_008988	
Software, algorithm	MaxChelator	Stanford University	RRID:SCR_003210	
Software, algorithm	Patchmaster version 2x90.2	HEKA, Multichannel Systems	RRID:SCR_000034	
Software, algorithm	Fluoview 300 version 4.3	Olympus	RRID:SCR_020339	
Software, algorithm	Mathematica 14	Wolfram	RRID:SCR_014448	
Software, algorithm	Igor Pro, version 6.32 and 8.03	WaveMetrics	RRID:SCR_000325	
Software, algorithm	Sigma Plot 11.0	Dundas Software	RRID:SCR_003210	
Other	Vibratome HM 650 V	Microm (Epredia), Fisher scientific	RRID:SCR_008452	Slicing device
Other	Borosilicate glass	Hilgenberg	Cat. # 1403569	Patch pipettes
Other	Puller PC-10	Narishige	RRID:SCR_022057	Pulling device
Other	Microscope BX51WI	Olympus	RRID:SCR_023069	Imaging device
Other	Amplifier EPC10/2	HEKA, Multichannel Systems	RRID:SCR_018399	Electrophysiology device
Other	Stimulus Isolator ISO-Stim 01 DPI	NPI electronics	RRID:SCR_016002	Electrophysiology device
Other	Fluoview-300 scanner	Olympus	RRID:SCR_020339	Imaging device
Other	Tsunami Ti:sapphire laser	Newport-Spectra Physics	https://www.spectra-physics.com	Imaging device
Other	Pockels cell 350–80 KD*P	Conoptics	https://www.conoptics.com	Imaging device
Other	Filters HC647/75, Semrock HC525/50, 720-SP	AHF	https://ahf.de	Imaging device
Other	PMT module H7422-40	Hamamatsu	https://www.hamamatsu.com RRID:SCR_017105	Imaging device
Other	PMT module PMT-02M/PMM-03	NPI electronics	RRID:SCR_016002	Imaging device

### Slice preparation

C57BL/6J mice at P21–26 and P90–100 were used in this study. Age-matched wild-type animals of both sexes were randomly allocated to respective treatment groups. All experiments were performed in accordance with the guidelines for the welfare of experimental animals issued by the European Communities Council Directive (2010/63/EU) and with the German Protection of Animals Act (Tierschutzgesetz). Mice were bred in the animal facility of the Medical Faculty of Leipzig University and were housed in individually ventilated cages in a specific pathogen-free environment and in a 12 hr/12 hr light-dark cycle with access to food and water ad libitum. Experiments were approved by the animal welfare office of the University Medical Center, Leipzig, as well as the local governmental authorities (Landesdirektion Leipzig, registration numbers T10/20, T05/21-MEZ) and reported in compliance with the ARRIVE guidelines.

C57BL/6J mice were decapitated under deep isoflurane (Curamed) inhalation anesthesia. The brain was excised rapidly and placed in cooled (0–4°C) ACSF containing (in mM): 125 NaCl, 2.5 KCl, 1.25 NaH_2_PO_4_, 26 NaHCO_3_, 1 MgCl_2_, 2 CaCl_2_, and 20 glucose, equilibrated with 95% O_2_ and 5% CO_2_ (pH 7.3–7.4). Coronar neocortical slices (150–250 μm thick) were cut from the lateral PFC, mPFC, or S1 region (Figure 1A) with a vibratome (HM 650 V, Microm). For some paired recordings between L5PNs and for calcium imaging experiments, parasagittal slices (200 µm) were prepared from the PFC region. Slices were incubated for 30 min at 35°C and subsequently stored at room temperature. For experiments, slices were transferred to a recording chamber and continuously perfused with ACSF (2–3 ml per min) supplemented with 10 μM (-)-bicuculline methiodide (Tocris) at 31–33°C. Unless stated otherwise, chemicals were from Sigma-Aldrich, Merck, and Roth, respectively.

### Electrophysiological recordings

Patch-clamp recordings from L5PNs located in the upper layer 5 (L5A in S1) were established according to the criteria described in detail in our previous work on this connection in S1 (Bornschein et al., 2019a; Bornschein et al., 2025). Presynaptic neurons were stimulated extracellularly in upper layer 2/3 (L2/3-L5PN connections) straight above the patched L5PN or in on-cell mode in L5A right next to the postsynaptic cell (L5PN-L5PN connections; Figure 1).

Patch pipettes were prepared from borosilicate glass (2.0/1.4 mm outer/inner diameter; Hilgenberg, Malsfeld, Germany) with a PC-10 puller (Narishige, London, UK) and had final resistances of 6–8 MΩ when filled with the following standard pipette solution (in mM): 150 K-gluconate, 4 NaCl, 3 MgCl_2_, 3 Na_2_ATP, 0.3 NaGTP, 0.05 EGTA, 10 KHEPES, dissolved in purified water. The pH was adjusted to 7.3 with KOH or HCl. Recordings were performed under optical control (BX51WI, Olympus, Hamburg, Germany), using an EPC10/2 amplifier and Patchmaster software (version v2x90.2, HEKA, Reutlingen, Germany). EPSCs were recorded in the whole-cell configuration at a holding potential (*V*_hold_) of –90 mV (online corrected for a liquid junction potential of 16 mV), filtered at 5 kHz and sampled at 10 kHz. Series resistance (R_s_) was continuously monitored and compensated to a fixed value between 10 and 15 MΩ. The average uncompensated R_s_ was 18±1 MΩ in L5PN-L5PN paired recordings (*n*=41 cells, mean ± SEM) and 23±1 MΩ in L2/3-L5PN recordings (*n*=133 cells). Holding current (*I*_hold_) was also monitored continuously and was on average –134±16 pA in L5PN-L5PNs and –172±11 in L2/3-L5PNs. Exclusion criteria were pre-established in previous studies on this connection (Bornschein et al., 2019a; Bornschein et al., 2025). Blinding was not performed during electrophysiological recordings because the primary experimental outcomes were objectively quantified according to clearly predefined parameters, thereby limiting the potential for observer bias.

In paired recordings, presynaptic L5PNs were stimulated in on-cell configuration (200–500 mV, 1–2 ms). In the chelator wash-in experiments, presynaptic neurons were repatched with a pipette solution supplemented with 10 mM EGTA (K-gluconate concentration was reduced to 135 mM to adjust osmolarity) and whole-cell configuration was established to allow EGTA perfusion of the presynaptic neuron. EPSCs were evoked every 10 s for at least 30 min (Figure 2). Averaged EPSC amplitudes were calculated before (≥10 min baseline) and 20–30 min after repatching (test period). For recordings from L2/3-L5PN connections, ACSF-filled patch pipettes were placed close to the somata of PNs in L2/3, and extracellular stimulation was performed using an ISO-Stim 01 DPI (NPI Electronics, Tamm, Germany). Minimal stimulation (0.5–5 V) was used and yielded EPSC amplitudes of ~40 pA (47±3 pA, *n*=131 pairs), making it likely that we investigated transmission mainly between single or few L2/3PNs and L5PNs in these experiments. Following 10 min of stable recordings in standard ACSF (baseline), the bath solution was exchanged for ACSF containing either 0.1% DMSO and 0.01% Pluronic (control solution, Molecular Probes), 10 µM EGTA-AM, or 10 µM BAPTA-AM (Invitrogen) in DMSO/Pluronic for 30 min (incubation period) and thereafter replaced by standard ACSF again for another 10 min (test period; Figure 2, Figure 2—figure supplement 1). Averaged EPSC amplitudes were calculated during baseline and test period. The time courses of chelator effects on EPSC amplitudes were analyzed by binning EPSC amplitudes within 2 min intervals of recording time to average amplitudes. Chelator effects were expressed as averaged EPSC amplitudes during test period normalized to the corresponding baseline values.

VGCCs were inhibited by bath application of ACSF containing a subsaturating concentration of 5 µM CdCl_2_ (Figure 5E and F). NMDA receptors were blocked by the selective antagonist APV (50 μM; Figure 1—figure supplement 2). For assessing the effects of the individual blockers, EPSCs were recorded in ACSF for at least 10 min (10 s intervals); subsequently, the blocker was perfused, and the blocker effects were quantified after a stable block had been established (at least 5 min test period).

### Quantification of quantal synaptic parameters

Quantal synaptic parameters were estimated from parabolic fits to mean-variance (MV) plots of EPSC amplitudes recorded at different [Ca^2+^]_e_ (1, 2, and 5 mM, ≥30 repetitions per concentration; [Mg^2+^]_e_ was correspondingly adjusted to 2, 1, 0 mM, respectively), assuming binomial release statistics (Clements and Silver, 2000; Scheuss et al., 2002; Silver, 2003; Figure 3). The recordings always started in 2 mM [Ca^2+^]_e_. The variance of EPSCs (*σ*^2^) was calculated according to Scheuss et al., 2002, plotted against the averaged amplitudes (*I*), and fitted by a parabola of the form:

$$\displaystyle \sigma^2 = {Iq} - \frac{{I}^2}{N}\left(1 + \mathrm{CV}_{\rm II}^{2}\right) + qI\mathrm{CV}_{\rm I}^{2} $$

where *q* is the quantal size, *N* a binomial parameter, and *CV*_I_ and *CV*_II_ the coefficients of intrasite and intersite quantal variability, respectively, assumed to be 0.3 (Clements and Silver, 2000). For CV assumptions that deviate from the standard value of 0.3, deviations in the calculated *p*_N_ values of less than 10% are to be expected (Schmidt et al., 2013; Bornschein et al., 2019a). The fits were constrained to pass through zero. The variance of the variance was calculated according to Meyer et al., 2001. Stationarity of EPSC amplitudes was established when, after full exchange of the bathing solutions (≥5 min), EPSC amplitudes no longer showed a tendency to increase or decline. For MV analysis in L2/3-L5PN connections, the bath solution was supplemented with the competitive AMPA receptor antagonist kynurenic acid (Kyn, 0.25 mM), which relieves their desensitization and saturation (Neher and Sakaba, 2001), and with 50 µM APV to prevent NMDAR activation. Due to the small size of EPSCs in L5PN-L5PN connections, no blocker was used in these recordings. The size of qEPSCs was quantified from evoked miniature EPSCs recorded in low [Ca^2+^]_e_ (1 mM) and high [Mg^2+^]_e_ (2 mM).

### Ca^2+^ imaging

Action potential-evoked (current injection of 1–4 nA for 1–2 ms) fluorescence changes were recorded at boutons located on axon collaterals of L5PNs as described previously (Bornschein et al., 2019a; Bornschein et al., 2025). L5PNs were filled with EGTA-free, Fluo-5F (200 µM, Invitrogen), and Atto-594 (50 µM, Atto-Tec) containing pipette solution via somatic whole-cell patch pipettes (Figure 4). Basal [Ca^2+^]_i_ values in S1 were additionally determined by OGB-1 (100 µM, Invitrogen; Figure 4—figure supplement 1). Cells were dialyzed for at least 20 min to yield sufficient equilibration with the dyes. Volume-averaged fluorescence signals were recorded in point mode at 500 kHz temporal resolution and subsequently binned to 500 Hz, using a custom-built two-photon microscope based on a Fluoview-300 scanner (Olympus), a 60×/0.9 N.A. objective, a mode-locked Ti:sapphire laser (Tsunami, Newport-Spectra Physics, Darmstadt, Germany; set to a center wavelength of 810 nm), and a Pockels cell (350–80 KD*P, Conoptics, Danbury, CT, USA). The fluorescence background was determined in a subsequent point mode recording performed near the boutons under investigation. On average, 11 (but at least 3) boutons per L5PN were recorded, and averages per cell were calculated to include a large number of putative presynapses. The fluorescence signals were filtered (HC647/75, Semrock HC525/50, 720-SP, AHF, Tübingen, Germany), detected by two external PMT modules (H7422-40, Hamamatsu, Herrsching am Ammersee, Germany; PMT-02M/PMM-03, NPI Electronics, Tamm, Germany) monitoring red and green epifluorescence, respectively, at fixed PMT voltages, and digitized with the Fluoview system. The Ca^2+^-dependent green fluorescence was normalized to the Ca^2+^-insensitive red fluorescence and expressed as background-corrected ∆*G*/*R* (Sabatini et al., 2002). ∆*G*/*R* signals were converted to changes in [Ca^2+^]_i_ based on an in vitro quantification of the *K*_D_ of Fluo-5F (439 nM) and OGB-1 (166 nM), respectively, in our pipette solution, adjusted with CaEGTA and K_2_EGTA (100 mM stock solutions, 10 mM HEPES added) to contain different free Ca^2+^ concentrations that were calculated with the MaxChelator. *R*_min_ and *R*_max_ values were measured after each successful recording in sealed patch pipettes that were placed in the slices near the recorded cells. The pipettes contained the EGTA-free, Fluo-5F- and Alexa-594-containing pipette solution with either 0 mM CaCl_2_ and 10 mM K_2_EGTA (zero Ca^2+^ solution) or 20 mM CaCl_2_ (high Ca^2+^ solution).

### Modeling

The kinetic gating scheme of the VGCCs and the reaction schemes for Ca^2+^ binding to buffers and transmitter release were converted to ordinary differential equations (ODEs). The system of ODEs was numerically solved using ‘NDSolve’ or ‘NDSolveValue’ of Mathematica 14 (Wolfram) as described previously (Schmidt et al., 2013; Bornschein et al., 2019a; Bornschein et al., 2025). Spatial resolution was achieved by placing the ODEs in concentric hemi-shells (1 or 2 nm thickness) that were coupled via diffusion.

The presynaptic action potential was modeled with a half-duration of 0.2 ms, *τ*_rise_ of 0.1, and *τ*_decay_ of 0.2 ms (Borst et al., 1995). Voltage-dependent activation of VGCCs was simulated using the gating scheme for P/Q-type channels, a single channel conductance of 2.5 pS, and a Ca^2+^ equilibrium potential of 130 mV (Li et al., 2007). The model included ATP as mobile buffer and immobile endogenous buffers with a buffer capacity (κ_E_) of 25 (Tran and Stricker, 2018). Model parameters are given in Table 2.

**Table 2.** Model parameters.

Parameters		Values	References/notes
Bouton volume		0.13 * 10^–15^ l	Schikorski and Stevens, 1999 *
Calcium	[Ca^2+^]_rest_	30*10^–9^ M	Measured
	*D*	220 *10^–12^ m^2^s^–1^	Allbritton et al., 1992
Fluo-5F	[Fluo]	140 *10^–6^ M	70% of pipette concentration
	*K*_D_	439 *10^–9^ M	Measured
	*k*_off_	300 s^–1^	Scott and Rusakov, 2006
	*D*	220 *10^–12^ m^2^s^–1^	^2^†
EGTA	*K*_D_	7*10^–8^ M	Klicken Sie hier, um Text einzugeben (Nägerl et al., 2000)
	*k*_off_	0.7 s^–1^	ibid.
	*k*_on_	1*10^7^ M^–1^s^–1^	ibid.
	*D*	220 *10^–12^ m^2^s^–1^	Naraghi and Neher, 1997
ATP	[ATP]	6 *10^–4^ M	Pipette concentration
	*K*_D_	2*10^–4^ M	Meinrenken et al., 2002
	*k*_off_	100 000 s^–1^	ibid.
	*k*_on_	5*10^8^ M^–1^s^–1^	ibid.
	*D*	220 *10^–12^ m^2^s^–1^	ibid.
	*κ*	3	Calculated
Immobile endogenous Ca^2+^ buffer (EB)	*κ*_E_	25	Tran and Stricker, 2018; Bornschein et al., 2019a
Fast EB	[EB_f_]	46.5 *10^–6^ M	Calculated
	*K*_D_	2*10^–6^ M	Vyleta and Jonas, 2014; Bornschein et al., 2019a
	*k*_off_	1000 s^–1^	ibid.
	*k*_on_	5*10^8^ M^–1^s^–1^	ibid.
	*κ*_E_	23.25	Calculated
Slow EB	[EB_s_]	1.23*10^–7^ M	Calculated
	*K*_D_	7*10^–8^ M	Nägerl et al., 2000
	*k*_off_	0.7 s^–1^	ibid.
	*k*_on_	1*10^7^ M^–1^s^–1^	ibid.
	*κ*_E_	1.75	Calculated
P/Q-type channel	*a*, *b*	247.71, 8.28 ms^–1^	Li et al., 2007
	*α*_1,0_,*β*_1,0_	5.89, 14.99 ms^–1^	ibid.
	*α*_2,0_, *β*_2,0_	9.21, 6.63 ms^–1^	ibid.
	*α*_3,0_, *β*_3,0_	5.2, 132.8 ms^–1^	ibid.
	*α*_4,0_,*β*_4,0_	1823.18, 248.58 ms^–1^	ibid.
	*k*1	62.61 mV	ibid.
	*k*2	33.92 mV	ibid.
	*k*3	135.08 mV	ibid.
	*k*4	20.86 mV	ibid.
Release sensor models			Bornschein et al., 2025
Syt1 model	*k*_off_	10 000 s^–1^	ibid.
	*k*_on_	1*10^8^ M^–1^s^–1^	ibid.
	*γ*	5900 s^–1^	ibid.
	*β*	4	ibid.
Syt1 priming model	*k*_off_	10 000 s^–1^	ibid.
	*k*_on_	1*10^8^ M^–1^s^–1^	ibid.
	*γ*	4000 s^–1^	ibid.
	*β*	4	ibid.
	*K*_M_	20*10^–6^ M	ibid.
	*k*_prim_	6000 s^–1^	ibid.
	*n*	5	ibid.

* Corrected for the space occupied by vesicles and organelles (Wilhelm et al., 2014).

† Assumed to be similar to free Ca^2+^ (Bucurenciu et al., 2008).

The model for each channel consisted of five closed (*C*) and one open (*O*) state. The transition between the first five steps was voltage-dependent and the last step voltage-independent:

(2) $$\displaystyle  C_0 \underset{\beta_1(V)}{\overset{\alpha_1(V)}{\rightleftharpoons}} C_1 \underset{\beta_2(V)}{\overset{\alpha_2(V)}{\rightleftharpoons}} C_2 \underset{\beta_3(V)}{\overset{\alpha_3(V)}{\rightleftharpoons}} C_3 \underset{\beta_4(V)}{\overset{\alpha_4(V)}{\rightleftharpoons}} C_4 \underset{b}{\overset{a}{\rightleftharpoons}} O$$

*a* and *b* are the rate constants for transitions between *C*_4_ and *O*, and *α_i_*(*V*), and *β_i_*(*V*) are the voltage dependent forward and backward transition rates that are given by:

(3a) $$\displaystyle \alpha_i(V) = \alpha_{i,0}\exp\left(\frac{V}{k_i}\right) \qquad i = 1-4$$

(3b) $$\displaystyle \beta_i(V) = \beta_{i,0}\exp\left(-\frac{V}{k_i}\right) \qquad i = 1-4$$

where *α_i,0_* and *β_i,0_* are the forward and backward rate constants at 0 mV and *k_i_* a slope factor.

Ca^2+^ binding to all buffers (*B*) was simulated by second-order kinetics with forward and backward binding rate constants *k*_on_ and *k*_off_, respectively:

(4a) $$\displaystyle \frac{d\left [Ca\right ]}{dt}=- k_{on}\left [Ca\right ]\left [B\right ]+k_{off}\left [CaB\right ]$$

(4b) $$\displaystyle \frac{d\left [B\right ]}{dt}=\frac{- d\left [CaB\right ]}{dt}$$

Diffusion of all species (*X*) was simulated according to

(5) $$\displaystyle \frac{d\left [X\right ]}{dt}=D_{x}\frac{A_{n,n\pm 1}}{V_{n}r}\left (\left [X\right ]_{n\pm 1}- \left [X\right ]_{n}\right)$$

where *D_X_* is the diffusion coefficient, *A* the surface, *V* the volume, and *r* the radius of a shell (*n*). Ca^2+^ was cleared by a linear, surface-based extrusion mechanism driven by the difference between [Ca^2+^]_i_ and the resting [Ca^2+^]_i_ (30 nM according to the imaging data).

**Scheme 1.**
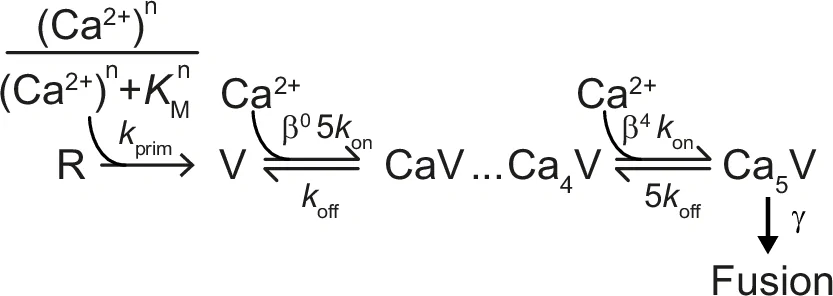
Syt1 priming model.

**Scheme 2.**
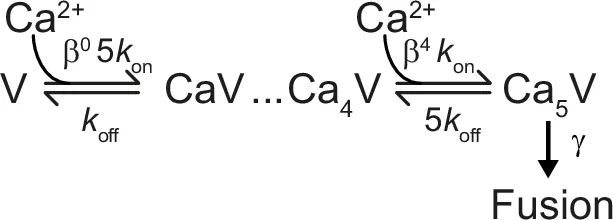
Syt1 model.

For fitting the model to the average experimental Ca^2+^ transient, the number of VGCCs was adjusted, yielding 33 VGCCs (Figure 5). The other free parameter of the simulation was the extrusion rate, which however affected only the later phase of the decay of the transient. During fitting, the model included the Ca^2+^ indicator dye. Fitting was first performed in a single compartment model (Helmchen and Tank, 2005). Subsequently, fitting was performed in the spatially resolved model with the numbers of VGCCs remaining unaltered and [Ca^2+^]_i_ as reported by the dye was calculated from the concentration of the Ca^2+^-dye complex assuming equilibrium conditions (Schmidt et al., 2003). Since L5PNs are characterized by an immobile and small *κ*_*E*_ with unknown binding kinetics, a slow (EB_s_) and a fast immobile endogenous buffer (EB_f_) were assumed (Helmchen et al., 1996; Ohana and Sakmann, 1998; Tran and Stricker, 2018). The fractions of EB_s_ and EB_f_ were adjusted to optimize the fit to the measured Ca^2+^ transient and to sum up to *κ*_*E*_ of 25. Following fitting, the indicator dye was removed from the model and the recently developed model for release from L5PN terminals was included (Bornschein et al., 2025). Either the ‘Syt1 priming model’ (Scheme 1; Figure 5) or the ‘Syt1 model’ (Scheme 2; Figure 5—figure supplement 1) were placed at varying distances from the Ca^2+^ sources.

In the priming model (Scheme 1), a single Ca^2+^-dependent priming step with Michaelis-Menten kinetics (*K*_M_) and a priming rate *k*_prim_ is assumed. *V* is the vesicular release sensor of a primed vesicle (Schemes 1 and 2) that binds a maximum of 5 Ca^2+^ ions. *k*_on_ and *k*_off_ are the forward and backward Ca^2+^ binding rate constants, *β*^*i*^ (*i*=0–4) is a cooperativity factor, and *γ* the fusion rate. *p*_N_ was obtained by integrating the release rates over time.

A ring-like arrangement of the VGCCs around a vesicle was assumed (Figure 5D; ring 1 model in Bornschein et al., 2019a). The number of VGCCs driving release was continuously reduced from the maximal value of 33 (Figure 5A), and the coupling distances at which the simulated *p*_N_ values matched the experimentally determined *p*_N_ were obtained. Subsequently, EGTA was included, and the consistency between the experimentally quantified EGTA effect and the simulated EGTA effect at a given coupling distance was determined (Figure 5B). The best match between experiments and simulation was obtained for nine VGCCs at a coupling distance of 51 nm (Figure 5C).

### Quantification and statistical analysis

Data from L5PN-L5PN pairs in PFC were compared to previously published data from these pairs in S1 (Table 1; Bornschein et al., 2019b; Bornschein et al., 2019a). Synaptic responses were determined by fitting a product of two exponential functions to the baseline-subtracted currents, which allows for independent adjustment of the time constants of the rising and falling phases, and minimizes noise effects (Bornschein et al., 2013). Synaptic delays were determined from the onset of stimulation to the fitted onset of the EPSC. PPRs were calculated by dividing the second amplitude of two consecutive EPSCs by the first.

Electrophysiological data and Ca^2+^ imaging data were analyzed using custom-written routines in Igor Pro (versions 6.32 and 8.03, WaveMetrics). Data are shown as median and interquartile range (IQR) or as mean ± standard error (SEM). Summarized data are either shown in boxplots as median ± IQR with mean values included as dashed lines and outliers as dots; or in bar graphs as mean ± SEM. Normality was tested using the Shapiro-Wilk test. Normally distributed data were compared with the t-test (two groups) or a one-way ANOVA (more than two groups). Non-normally distributed or small samples of data were compared with the Mann-Whitney U rank sum test (MWU; two groups) or a Kruskal-Wallis ANOVA on ranks (more than two groups). For multiple comparisons, post hoc testing was performed with the Holm-Sidak (one-way ANOVA) or Dunn’s method (ANOVA on ranks). To compare pre- and post-treatment data, the paired t-test or the Wilcoxon signed-rank test (WSR) was used, depending on the distribution of the data. All statistical tests were two-tailed. p-Values are indicated as *p<0.05, **p<0.01, and ***p<0.001. The number of experiments *n* represents the number of biological replicates, i.e., the number of cell pairs, cells, or boutons, and was chosen sufficiently high to permit reliable statistical analysis. All of the statistical details of experiments can be found in the figures and corresponding figure legends. Statistics were performed with Sigma Plot 11.0 (Dundas Software) and Mathematica 14 (Wolfram).

## Data Availability

All data are available in the main text or in the supplemental information.All original code has been deposited at GitHub and is publicly available (https://github.com/Hartmut-Schmidt/eLife2026, copy archived at Hartmut-Schmidt, 2026).

## Additional files

MDAR checklist

## References

[bib1] Adler EM, Augustine GJ, Duffy SN, Charlton MP (1991). Alien intracellular calcium chelators attenuate neurotransmitter release at the squid giant synapse. The Journal of Neuroscience.

[bib2] Allbritton NL, Meyer T, Stryer L (1992). Range of messenger action of calcium ion and inositol 1,4,5-trisphosphate. Science.

[bib3] Baur D, Bornschein G, Althof D, Watanabe M, Kulik A, Eilers J, Schmidt H (2015). Developmental tightening of cerebellar cortical synaptic influx-release coupling. The Journal of Neuroscience.

[bib4] Beaulieu C (1993). Numerical data on neocortical neurons in adult rat, with special reference to the GABA population. Brain Research.

[bib5] Blatow M, Rozov A, Katona I, Hormuzdi SG, Meyer AH, Whittington MA, Caputi A, Monyer H (2003). A novel network of multipolar bursting interneurons generates theta frequency oscillations in neocortex. Neuron.

[bib6] Bollmann JH, Sakmann B, Borst JG (2000). Calcium sensitivity of glutamate release in a calyx-type terminal. Science.

[bib7] Bornschein G, Arendt O, Hallermann S, Brachtendorf S, Eilers J, Schmidt H (2013). Paired-pulse facilitation at recurrent Purkinje neuron synapses is independent of calbindin and parvalbumin during high-frequency activation. The Journal of Physiology.

[bib8] Bornschein G, Eilers J, Schmidt H (2019a). Neocortical high probability release sites are formed by distinct Ca^2+^ channel-to-release sensor topographies during development. Cell Reports.

[bib9] Bornschein G, Brachtendorf S, Schmidt H (2019b). Developmental increase of neocortical presynaptic efficacy *via* maturation of vesicle replenishment. Frontiers in Synaptic Neuroscience.

[bib10] Bornschein G, Schmidt H (2019c). Synaptotagmin Ca^2+^ sensors and their spatial coupling to presynaptic cav channels in central cortical synapses. Frontiers in Molecular Neuroscience.

[bib11] Bornschein G, Brachtendorf S, Reinert A, Eshra A, Kraft R, Hirrlinger J, Eilers J, Hallermann S, Schmidt H (2025). The intracellular Ca^2+^ sensitivity of transmitter release in glutamatergic neocortical boutons. Science.

[bib12] Borst JG, Helmchen F, Sakmann B (1995). Pre- and postsynaptic whole-cell recordings in the medial nucleus of the trapezoid body of the rat. The Journal of Physiology.

[bib13] Brachtendorf S, Bornschein G, Schmidt H (2025). Estimates of quantal synaptic parameters in light of more complex vesicle pool models. Frontiers in Cellular Neuroscience.

[bib14] Brandt A, Khimich D, Moser T (2005). Few Ca_V_1.3 channels regulate the exocytosis of a synaptic vesicle at the hair cell ribbon synapse. The Journal of Neuroscience.

[bib15] Bucurenciu I, Kulik A, Schwaller B, Frotscher M, Jonas P (2008). Nanodomain coupling between Ca^2+^ channels and Ca^2+^ sensors promotes fast and efficient transmitter release at a cortical GABAergic synapse. Neuron.

[bib16] Bullmann T, Kaas T, Ritzau-Jost A, Wöhner A, Kirmann T, Rizalar FS, Holzer M, Nerlich J, Puchkov D, Geis C, Eilers J, Kittel RJ, Arendt T, Haucke V, Hallermann S (2024). Human iPSC-derived neurons with reliable synapses and large presynaptic action potentials. The Journal of Neuroscience.

[bib17] Castillo PE (2012). Presynaptic LTP and LTD of excitatory and inhibitory synapses. Cold Spring Harbor Perspectives in Biology.

[bib18] Chanda S, Xu-Friedman MA (2010). A low-affinity antagonist reveals saturation and desensitization in mature synapses in the auditory brain stem. Journal of Neurophysiology.

[bib19] Chen JJ, Kaufmann WA, Chen C, Arai I, Kim O, Shigemoto R, Jonas P (2024). Developmental transformation of Ca^2+^ channel-vesicle nanotopography at a central GABAergic synapse. Neuron.

[bib20] Clements JD, Silver RA (2000). Unveiling synaptic plasticity: a new graphical and analytical approach. Trends in Neurosciences.

[bib21] Douglas RJ, Martin KAC (2004). Neuronal circuits of the neocortex. Annual Review of Neuroscience.

[bib22] Doussau F, Schmidt H, Dorgans K, Valera AM, Poulain B, Isope P (2017). Frequency-dependent mobilization of heterogeneous pools of synaptic vesicles shapes presynaptic plasticity. eLife.

[bib23] Eggermann E, Bucurenciu I, Goswami SP, Jonas P (2012). Nanodomain coupling between Ca2+ channels and sensors of exocytosis at fast mammalian synapses. Nature Reviews Neuroscience.

[bib24] Fedchyshyn MJ, Wang LY (2005). Developmental transformation of the release modality at the calyx of Held synapse. The Journal of Neuroscience.

[bib25] Feldman DE (2009). Synaptic mechanisms for plasticity in neocortex. Annual Review of Neuroscience.

[bib26] Feldman DE (2012). The spike-timing dependence of plasticity. Neuron.

[bib27] Feldmeyer D, Radnikow G (2009). Developmental alterations in the functional properties of excitatory neocortical synapses. The Journal of Physiology.

[bib28] Feldmeyer D (2012). Excitatory neuronal connectivity in the barrel cortex. Frontiers in Neuroanatomy.

[bib29] Felmy F, Neher E, Schneggenburger R (2003). Probing the intracellular calcium sensitivity of transmitter release during synaptic facilitation. Neuron.

[bib30] Frick A, Feldmeyer D, Helmstaedter M, Sakmann B (2008). Monosynaptic connections between pairs of L5A pyramidal neurons in columns of juvenile rat somatosensory cortex. Cerebral Cortex.

[bib31] Harlow ML, Ress D, Stoschek A, Marshall RM, McMahan UJ (2001). The architecture of active zone material at the frog’s neuromuscular junction. Nature.

[bib32] Harris KD, Shepherd GMG (2015). The neocortical circuit: themes and variations. Nature Neuroscience.

[bib33] (2026). Software Heritage.

[bib34] Hefft S, Kraushaar U, Geiger JRP, Jonas P (2002). Presynaptic short-term depression is maintained during regulation of transmitter release at a GABAergic synapse in rat hippocampus. The Journal of Physiology.

[bib35] Helmchen F, Imoto K, Sakmann B (1996). Ca^2+^ buffering and action potential-evoked Ca^2+^ signaling in dendrites of pyramidal neurons. Biophysical Journal.

[bib36] Helmchen F, Tank DW, Yuste R, Konnerth A (2005). Imaging Neurons: A Laboratory Manual.

[bib37] Iwasaki S, Takahashi T (2001). Developmental regulation of transmitter release at the calyx of Held in rat auditory brainstem. The Journal of Physiology.

[bib38] Jackman SL, Regehr WG (2017). The mechanisms and functions of synaptic facilitation. Neuron.

[bib39] Jarsky T, Tian M, Singer JH (2010). Nanodomain control of exocytosis is responsible for the signaling capability of a retinal ribbon synapse. The Journal of Neuroscience.

[bib40] Keller D, Babai N, Kochubey O, Han Y, Markram H, Schürmann F, Schneggenburger R (2015). An exclusion zone for Ca^2+^ channels around docked vesicles explains release control by multiple channels at a CNS synapse. PLOS Computational Biology.

[bib41] Koike-Tani M, Kanda T, Saitoh N, Yamashita T, Takahashi T (2008). Involvement of AMPA receptor desensitization in short-term synaptic depression at the calyx of Held in developing rats. The Journal of Physiology.

[bib42] Kroon T, van Hugte E, van Linge L, Mansvelder HD, Meredith RM (2019). Early postnatal development of pyramidal neurons across layers of the mouse medial prefrontal cortex. Scientific Reports.

[bib43] Kusch V, Bornschein G, Loreth D, Bank J, Jordan J, Baur D, Watanabe M, Kulik A, Heckmann M, Eilers J, Schmidt H (2018). Munc13-3 is required for the developmental localization of Ca^2+^ channels to active zones and the nanopositioning of Ca_v_2.1 near release sensors. Cell Reports.

[bib44] Li L, Bischofberger J, Jonas P (2007). Differential gating and recruitment of P/Q-, N-, and R-type Ca^2+^ channels in hippocampal mossy fiber boutons. The Journal of Neuroscience.

[bib45] Lin KH, Taschenberger H, Neher E (2022). A sequential two-step priming scheme reproduces diversity in synaptic strength and short-term plasticity. PNAS.

[bib46] Markram H, Lübke J, Frotscher M, Roth A, Sakmann B (1997). Physiology and anatomy of synaptic connections between thick tufted pyramidal neurones in the developing rat neocortex. The Journal of Physiology.

[bib47] Matthews G, Fuchs P (2010). The diverse roles of ribbon synapses in sensory neurotransmission. Nature Reviews Neuroscience.

[bib48] Matveev V, Zucker RS, Sherman A (2004). Facilitation through buffer saturation: constraints on endogenous buffering properties. Biophysical Journal.

[bib49] Meinrenken CJ, Borst JGG, Sakmann B (2002). Calcium secretion coupling at calyx of Held governed by nonuniform channel-vesicle topography. The Journal of Neuroscience.

[bib50] Meyer AC, Neher E, Schneggenburger R (2001). Estimation of quantal size and number of functional active zones at the calyx of Held synapse by nonstationary EPSC variance analysis. The Journal of Neuroscience.

[bib51] Meyer HS, Schwarz D, Wimmer VC, Schmitt AC, Kerr JND, Sakmann B, Helmstaedter M (2011). Inhibitory interneurons in a cortical column form hot zones of inhibition in layers 2 and 5A. PNAS.

[bib52] Miki T, Malagon G, Pulido C, Llano I, Neher E, Marty A (2016). Actin- and myosin-dependent vesicle loading of presynaptic docking sites prior to exocytosis. Neuron.

[bib53] Moser T, Beutner D (2000). Kinetics of exocytosis and endocytosis at the cochlear inner hair cell afferent synapse of the mouse. PNAS.

[bib54] Nägerl UV, Novo D, Mody I, Vergara JL (2000). Binding kinetics of calbindin-D(28k) determined by flash photolysis of caged Ca(2+). Biophysical Journal.

[bib55] Nakamura Y, Harada H, Kamasawa N, Matsui K, Rothman JS, Shigemoto R, Silver RA, DiGregorio DA, Takahashi T (2015). Nanoscale distribution of presynaptic Ca(2+) channels and its impact on vesicular release during development. Neuron.

[bib56] Naraghi M, Neher E (1997). Linearized buffered Ca^2+^ diffusion in microdomains and its implications for calculation of [Ca^2+^] at the mouth of a calcium channel. The Journal of Neuroscience.

[bib57] Narayanan NS, Hyman JM, Seamans J, Rich EL (2025). Computational properties of the prefrontal cortex. The Journal of Neuroscience.

[bib58] Neher E, Sakaba T (2001). Combining deconvolution and noise analysis for the estimation of transmitter release rates at the calyx of held. The Journal of Neuroscience.

[bib59] Neher E, Brose N (2018). Dynamically primed synaptic vesicle states: key to understand synaptic short-term plasticity. Neuron.

[bib60] Neher E (2024). Interpretation of presynaptic phenotypes of synaptic plasticity in terms of a two-step priming process. The Journal of General Physiology.

[bib61] Ohana O, Sakmann B (1998). Transmitter release modulation in nerve terminals of rat neocortical pyramidal cells by intracellular calcium buffers. The Journal of Physiology.

[bib62] Rebola N, Reva M, Kirizs T, Szoboszlay M, Lőrincz A, Moneron G, Nusser Z, DiGregorio DA (2019). Distinct nanoscale calcium channel and synaptic vesicle topographies contribute to the diversity of synaptic function. Neuron.

[bib63] Regehr WG, Carey MR, Best AR (2009). Activity-dependent regulation of synapses by retrograde messengers. Neuron.

[bib64] Reyes A, Sakmann B (1999). Developmental switch in the short-term modification of unitary EPSPs evoked in layer 2/3 and layer 5 pyramidal neurons of rat neocortex. The Journal of Neuroscience.

[bib65] Rozov A, Burnashev N, Sakmann B, Neher E (2001). Transmitter release modulation by intracellular Ca^2+^ buffers in facilitating and depressing nerve terminals of pyramidal cells in layer 2/3 of the rat neocortex indicates a target cell-specific difference in presynaptic calcium dynamics. The Journal of Physiology.

[bib66] Sabatini BL, Oertner TG, Svoboda K (2002). The life cycle of Ca(2+) ions in dendritic spines. Neuron.

[bib67] Scheuss V, Schneggenburger R, Neher E (2002). Separation of presynaptic and postsynaptic contributions to depression by covariance analysis of successive EPSCs at the calyx of Held synapse. The Journal of Neuroscience.

[bib68] Schikorski T, Stevens CF (1999). Quantitative fine-structural analysis of olfactory cortical synapses. PNAS.

[bib69] Schmidt H, Stiefel KM, Racay P, Schwaller B, Eilers J (2003). Mutational analysis of dendritic Ca^2+^ kinetics in rodent Purkinje cells: role of parvalbumin and calbindin D28k. The Journal of Physiology.

[bib70] Schmidt H, Brachtendorf S, Arendt O, Hallermann S, Ishiyama S, Bornschein G, Gall D, Schiffmann SN, Heckmann M, Eilers J (2013). Nanodomain coupling at an excitatory cortical synapse. Current Biology.

[bib71] Schmidt H (2019). Control of presynaptic parallel fiber efficacy by activity-dependent regulation of the number of occupied release sites. Frontiers in Systems Neuroscience.

[bib72] Schneggenburger R, Neher E (2000). Intracellular calcium dependence of transmitter release rates at a fast central synapse. Nature.

[bib73] Scimemi A, Diamond JS (2012). The number and organization of Ca^2+^ channels in the active zone shapes neurotransmitter release from Schaffer collateral synapses. The Journal of Neuroscience.

[bib74] Scott R, Rusakov DA (2006). Main determinants of presynaptic Ca^2+^ dynamics at individual mossy fiber-CA3 pyramidal cell synapses. The Journal of Neuroscience.

[bib75] Shahrezaei V, Cao A, Delaney KR (2006). Ca^2+^ from one or two channels controls fusion of a single vesicle at the frog neuromuscular junction. The Journal of Neuroscience.

[bib76] Silver RA (2003). Estimation of nonuniform quantal parameters with multiple-probability fluctuation analysis: theory, application and limitations. Journal of Neuroscience Methods.

[bib77] Singer JH, Diamond JS (2003). Sustained Ca^2+^ entry elicits transient postsynaptic currents at a retinal ribbon synapse. The Journal of Neuroscience.

[bib78] Taschenberger H, Leão RM, Rowland KC, Spirou GA, von Gersdorff H (2002). Optimizing synaptic architecture and efficiency for high-frequency transmission. Neuron.

[bib79] Tran V, Stricker C (2018). Diffusion of Ca^2+^ from small boutons en passant into the axon shapes AP-evoked Ca^2+^ transients. Biophysical Journal.

[bib80] Vyleta NP, Jonas P (2014). Loose coupling between Ca^2+^ channels and release sensors at a plastic hippocampal synapse. Science.

[bib81] Weichard I, Taschenberger H, Gsell F, Bornschein G, Ritzau-Jost A, Schmidt H, Kittel RJ, Eilers J, Neher E, Hallermann S, Nerlich J (2023). Fully-primed slowly-recovering vesicles mediate presynaptic LTP at neocortical neurons. PNAS.

[bib82] Wilhelm BG, Mandad S, Truckenbrodt S, Kröhnert K, Schäfer C, Rammner B, Koo SJ, Claßen GA, Krauss M, Haucke V, Urlaub H, Rizzoli SO (2014). Composition of isolated synaptic boutons reveals the amounts of vesicle trafficking proteins. Science.

[bib83] Yarim A, Brachtendorf S, Schmidt H, Bornschein G (2026). Pyramidal neuron synapses in M2 exhibit properties intermediate between prefrontal cortex and M1 synapses. bioRxiv.

[bib84] Zucker RS, Regehr WG (2002). Short-term synaptic plasticity. Annual Review of Physiology.

