# Supplementary material for: Loose coupling between Ca2+ channels and release sensors as a synaptic correlate of higher-order brain function
Source: eLife. 2026 Oct 28;15:RP110314. doi: 10.7554/eLife.110314 (PMC13719166; doi:10.7554/eLife.110314)
Supplement: Peer review history

# Editor report: eLife Assessment

**DOI:** 10.7554/eLife.110314.3.sa0

**Authors:** Annalisa Scimemi

**Affiliations:** University at Albany, State University of New York United States

**Keywords:** Convincing, Important

By using a combination of patch clamp recordings, calcium imaging and computer modeling, the authors analyze the spatial distribution of voltage gated calcium channels at glutamatergic synapses formed between layer 5 pyramidal neurons (L5PNs) and between layer 2/3 and L5PNs in the prefrontal cortex (PFC) and primary somatosensory cortex (S1); they conclude that the calcium channel-vesicle coupling is looser in the PFC compared to S1, and future ultrastructural studies may directly confirm the proposed differences in the spatial organization of voltage-gated calcium channels and vesicular release sites. Overall, these findings are **important** because they have implications for shaping synaptic plasticity and neural circuit function across brain regions. They are **convincing** because they are based on the use of a multi-pronged approach, although the presentation would benefit from stronger integration of the current findings with the existing literature and a more explicit discussion of potential limitations and confounding factors for data interpretation.

# Referee report: Reviewer #1 (Public review)

**DOI:** 10.7554/eLife.110314.3.sa1

Summary:

This study asks whether synapses formed by the same broad neuronal class (excitatory pyramidal neurons, PN) adapt their presynaptic organization in a cortex-specific manner, comparing prefrontal cortex (PFC) with primary somatosensory cortex (S1). The authors combine sophisticated electrophysiology (paired recordings and extracellular minimal stimulation), pharmacological perturbations of presynaptic Ca²⁺-secretion coupling, bouton Ca²⁺ imaging, and mechanistic modeling. Across two prominent excitatory connections (Layer 5 (L5) PN-L5PN and L2/3-L5PN), they provide convergent evidence that mature PFC synapses operate with looser Ca²⁺ channel-release sensor coupling than their S1 counterparts.

Overall, the study provides an appealing mechanistic link between synaptic nano/micro-architecture and cortical-area specialization. The idea that PFC synapses retain a more "plasticity-favoring" presynaptic state, while primary sensory cortex emphasizes reliability and timing precision, is potentially impactful for how we think about circuit computation and plasticity across cortical hierarchies.

Strengths:

A major strength is the multi-pronged experimental strategy. The paper first establishes robust, area-dependent differences in synaptic efficacy, reliability, timing, and short-term plasticity (facilitation prevailing in PFC versus depression in S1), using both paired recordings and minimal extracellular stimulation paradigms. The coupling interpretation is then directly supported by differential sensitivity to EGTA (and appropriate positive-control effects of fast chelators). Finally, volume averaged calcium signals are reported to be similar across areas, arguing against trivial explanations based on gross differences in calcium influx, and the modeling provides a quantitative framework for interpreting the observed chelator effects.

Weaknesses:

Limitations are minor and concern interpretation/clarity rather than core results. Some key inferences rely on indirect readouts (chelator sensitivity, fluctuation analysis-derived parameters, bouton-averaged calcium signals), each of which carries assumptions and potential confounds that should be discussed more explicitly. In particular, the, repatching paradigm for the paired-recording EGTA experiment, though very impressive, and the limited number of extracellular calcium conditions used for fluctuation analysis (three concentrations) can influence quantitative estimates and the confidence intervals around them.

# Referee report: Reviewer #2 (Public review)

**DOI:** 10.7554/eLife.110314.3.sa2

Schwarze et al. investigated whether synaptic efficacy is brain-region specific. To this end, they compared synaptic connections established by layer 5 (L5) neocortical pyramidal cells and between L5 and L2/3 pyramidal cells. In order to identify the mechanism of this brain region specificity, the authors employed several experimental approaches, including paired electrophysiological recordings, extracellular stimulation, low- and high-affinity intracellular calcium chelators (EGTA and BAPTA), multiple probability fluctuation analysis (MPFA), and intracellular measurements of calcium transients as well as computational modelling. The findings of the present study indicate that synaptic connections in the primary somatosensory cortex (S1) are significantly stronger and more reliable than those in the prefrontal cortex (PFC).

The study is timely and the topic is of significant interest to the neuroscience community. Despite the extensive research that has been carried out on the neuroanatomy and receptor distribution of different brain regions, comparatively little attention has been paid to differences in synaptic physiology. The authors' approach is characterised by its elegance and comprehensive nature, and the conclusions drawn are compelling.

Comments on revised manuscript:

I have no further issues with the present version of the manuscript. All my concerns and/or recommendations were satisfactorily addressed.

# Referee report: Reviewer #3 (Public review)

**DOI:** 10.7554/eLife.110314.3.sa3

Summary:

In this manuscript, Max Schwarze and colleagues examined the coupling distance between presynaptic Ca²⁺ channels and the vesicular release sensor at neocortical synapses in mouse. They propose that Ca²⁺ channel-release sensor coupling differs across cortical areas, with relatively loose (microdomain) coupling in prefrontal cortex (PFC) and tighter (nanodomain) coupling in primary somatosensory cortex (S1) for comparable pyramidal-neuron synapse types. To test this, they combine paired recordings and minimal stimulation with chelator manipulations (EGTA/BAPTA), mean-variance/MPFA-style analyses, presynaptic Ca²⁺ imaging, and computational modeling. They conclude that presynaptic coupling organization is area-specific in the mature cortex and contributes to regional differences in synaptic timing, reliability, and short-term plasticity.

Strengths:

This study tackles an important question and is strengthened by a cohesive body of evidence assembled from multiple complementary approaches. A major asset is the inclusion of high-value datasets, particularly the paired recordings between L5 pyramidal neurons and the systematic assessment of EGTA sensitivity, which provide a solid functional foundation for the authors' central claims. The work is further distinguished by its genuinely multimodal design: combining electrophysiology with presynaptic calcium imaging (and integrating these observations with quantitative analyses and modeling) offers a more mechanistic view of neurotransmitter release than any single method could provide. Overall, the direct, within-framework comparison of presynaptic release-control mechanisms across cortical areas for comparable synapse types is compelling and gives the conclusions a level of robustness and interpretability that is often difficult to achieve in studies of cortical synaptic diversity.

Weaknesses:

The principal limitation is incomplete cellular and synaptic specificity in parts of the study. The L2/3-L5PN experiments rely on minimal extracellular stimulation and therefore do not unambiguously identify the presynaptic neuron, its subtype or the number of recruited axons. Similarly, calcium imaging was performed at boutons on L5PN axon collaterals without identifying their postsynaptic targets. The imaging measurements could therefore combine boutons contacting pyramidal neurons and interneurons, potentially obscuring target-dependent differences in presynaptic calcium regulation. Recent connectomic studies demonstrate that local L5 pyramidal-cell axons can distribute substantial fractions of their output to inhibitory neurons, although the exact proportions depend strongly on pyramidal-cell subtype and distance along the axon.

The quantitative coupling-distance estimate is also model-dependent. The approximately 50-nm estimate for PFC synapses follows from a particular ring-like VGCC geometry and release-sensor model. The simulations demonstrate that this configuration is compatible with the data, but they do not uniquely identify the underlying molecular architecture.

Overall, the experiments support the narrower conclusion that the examined PFC and S1 synapses differ in functional Ca²⁺-channel-release-sensor coupling. The associated differences in synaptic timing, efficacy and plasticity are compelling, although coupling distance is not isolated causally from other regional differences in release-site number and quantal properties. The proposal that loose coupling is a general correlate of higher-order cortical function remains an interesting but currently speculative interpretation. Further comparisons across additional cortical regions and genetically or projection-defined synapse types will be particularly helpful in establishing the broader generality of this concept

Comments on revised version.

The authors have addressed most of my comments in the revised manuscript. I have only one remaining, relatively minor suggestion concerning point 5. I appreciate that the authors now acknowledge the possibility that the imaged boutons may contact different postsynaptic targets. However, the argument that interneuron-targeting boutons are likely to make only a minor contribution, based on the overall proportions of excitatory neurons or inhibitory synapses in the cortex, may not fully resolve this concern. Excitatory pyramidal neurons can distribute their outputs non-randomly across excitatory and inhibitory targets, and this distribution may depend on pyramidal-cell subtype and axonal distance. For example, a recent MICrONS/Allen Institute connectomic analysis of L5 extratelencephalic neurons in mouse visual cortex found that approximately two-thirds of their proximal synaptic outputs contacted inhibitory neurons. The proportion was close to 80% near the soma and decreased progressively with distance along the axon.

These findings concern a specific L5 pyramidal-cell subtype in visual cortex and therefore cannot be transferred directly to the PFC and S1 preparations examined here. Nevertheless, they illustrate that the postsynaptic target distribution of L5 pyramidal-neuron boutons cannot necessarily be inferred from the overall abundance of excitatory and inhibitory neurons or synapses.

# Author comment: Author response

**DOI:** 10.7554/eLife.110314.3.sa4

**Authors:** Grit Bornschein, Max Schwarze, Antonia Brunner, Akanksha Arshia, Simone Brachtendorf, Hartmut Schmidt

**Affiliations:** Leipzig University Leipzig Germany; Leipzig University Leipzig Germany; Leipzig University Leipzig Germany; Leipzig University Leipzig Germany; Leipzig University Leipzig Germany; Leipzig University Leipzig Germany

The following is the authors’ response to the original reviews.

**Public Reviews:**

**Reviewer #1 (Public review):**

Summary:

This study asks whether synapses formed by the same broad neuronal class (excitatory pyramidal neurons, PN) adapt their presynaptic organization in a cortex-specific manner, comparing the prefrontal cortex (PFC) with the primary somatosensory cortex (S1). The authors combine sophisticated electrophysiology (paired recordings and extracellular minimal stimulation), pharmacological perturbations of presynaptic Ca^2+^-secretion coupling, bouton Ca^2+^ imaging, and mechanistic modeling. Across two prominent excitatory connections (Layer 5 (L5) PN-L5PN and L2/3-L5PN), they provide convergent evidence that mature PFC synapses operate with looser Ca^2+^ channel-release sensor coupling than their S1 counterparts.

Overall, the study provides an appealing mechanistic link between synaptic nano/micro-architecture and cortical-area specialization. The idea that PFC synapses retain a more "plasticity-favoring" presynaptic state, while the primary sensory cortex emphasizes reliability and timing precision, is potentially impactful for how we think about circuit computation and plasticity across cortical hierarchies.

Strengths:

A major strength is the multi-pronged experimental strategy. The paper first establishes robust, area-dependent differences in synaptic efficacy, reliability, timing, and short-term plasticity (facilitation prevailing in PFC versus depression in S1), using both paired recordings and minimal extracellular stimulation paradigms. The coupling interpretation is then directly supported by differential sensitivity to EGTA (and appropriate positive-control effects of fast chelators). Finally, volume-averaged calcium signals are reported to be similar across areas, arguing against trivial explanations based on gross differences in calcium influx, and the modeling provides a quantitative framework for interpreting the observed chelator effects.

Weaknesses:

Limitations are minor and concern interpretation/clarity rather than core results. Some key inferences rely on indirect readouts (chelator sensitivity, fluctuation analysis-derived parameters, bouton-averaged calcium signals), each of which carries assumptions and potential confounds that should be discussed more explicitly. In particular, the repatching paradigm for the paired-recording EGTA experiment, though very impressive, and the limited number of extracellular calcium conditions used for fluctuation analysis (three concentrations), can influence quantitative estimates and the confidence intervals around them.

We would like to thank the reviewer for his/her overall positive assessment of our manuscript and the constructive advice, which helped us to improve our manuscript. We discussed the limitations, assumptions and potential confounding factors in more detail. We addressed them pointwise in the recommendations for the authors.

**Reviewer #2 (Public review):**

Schwarze et al. investigated whether synaptic efficacy is brain-region specific. To this end, they compared synaptic connections established by layer 5 (L5) neocortical pyramidal cells and between L5 and L2/3 pyramidal cells. In order to identify the mechanism of this brain region specificity, the authors employed several experimental approaches, including paired electrophysiological recordings, extracellular stimulation, low- and high-affinity intracellular calcium chelators (EGTA and BAPTA), multiple probability fluctuation analysis (MPFA), and intracellular measurements of calcium transients as well as computational modelling. The findings of the present study indicate that synaptic connections in the primary somatosensory cortex (S1) are significantly stronger and more reliable than those in the prefrontal cortex (PFC).

The study is timely, and the topic is of significant interest to the neuroscience community. Despite the extensive research that has been carried out on the neuroanatomy and receptor distribution of different brain regions, comparatively little attention has been paid to differences in synaptic physiology. The authors' approach is characterised by its elegance and comprehensive nature, and the conclusions drawn are compelling. Nevertheless, there are a number of unresolved issues.

First, we would like to thank the reviewer for his/her detailed survey of our work, which was very helpful in improving our manuscript. We are happy about the overall positive evaluation and the constructive comments. To fully clarify all points, we performed new experiments and analyses, in particular we determined EGTA sensitivity in PFC and S1 from the same animal and we performed MPFA with an additional extracellular Ca^2+^ concentration. We extended the discussion on the examined cell types. Overall, we carefully revised the manuscript to address all points. Please see below our point-wise response.

Major points:

(1) The authors state that data from the S1 cortex were obtained in a previous study. In the context of an explicitly comparative study (PFC vs. S1cortex), it would have been advantageous for the authors to perform a subset of experiments in which both cortices were obtained from a single animal. This is a feasible undertaking, given the spatial separation of the PFC and S1 cortex.

This is only true for the paired recordings from L5PN-L5PN connections in S1, which were obtained in a previous study and partially reanalyzed. All recordings from L2/3-L5PN connections in S1 and PFC as well as the paired recordings on L5PN-L5PN synapses in PFC were obtained in the present study. To make this clearer, we have added Table 1. This lists which data and associated figures are from this study and which are from previous studies (Bornschein et al., Cell Rep. 2019; Bornschein et al., Front. Syn. Neurosci. 2019).

Our experiments are lengthy and therefore it is challenging to achieve two successful recordings within the lifetime of acute brain slices. For this reason, the previous version of the manuscript did not include recordings from PFC and S1 of the same animal. We have now measured EGTA effects in L2/3-L5PNs from S1 and PFC of the same animal. Two new recordings were added to the EGTA-AM plots in Figure 2C-F and in the results section. Example recordings are shown in Figure S3A and B, as is the comparison of EGTA-AM effects in L2/3-L5PNs from PFC and S1 in Figure S3C, with data points from the same animal marked.

“On the other hand, EGTA significantly reduced EPSC amplitudes only in PFC (0.54, 0.47-0.67; 55% of control) but not in S1 (0.85, 0.81-1.04; 100% of control). For a better comparison of EGTA effects some recordings were performed in PFC and S1 derived from the same animal to rule out interindividual effects (see example recording in Figure S3A-C).”

(2) Figure 1A is somewhat misleading because it could suggest that the authors have performed dual recordings in identified PFC pyramidal cells.

We thank the reviewier for this helpful note. We added “L2/3 or L5” to the stimulation panel of Figure 1A to illustrate that we stimulated either extracellularly in L2/3 or L5PNs directly via the patch pipette.

(3) PFC and S1 cortex in rodents differ markedly in their morphological organisation. For example, in all sensory cortices, layer 4 is very pronounced; however, in the PFC of rodent,s no clear layer 4 can be found. On the other hand, PFC shows a clear separation of layers 2 and 3, which is not visible inthe S1 cortex. Furthermore, PFC pyramidal cells in layers 2, 3, and 5 exhibit significant heterogeneity, diverging considerably from those found in layers 5a and 5b of S1 cortex. Thus, there is no clear correlation between L5 pyramidal cells in the PFC and the S1 cortex. In order to achieve a meaningful comparison of the data obtained in PFC and S1 cortex, it is necessary for the authors to determine whether the record is from similar pyramidal cell populations.

(3) In addition, PFC pyramidal cells in layer 2, 3 and 5 are highly heterogeneous and differ markedly from those in layer 5a and 5b of S1 cortex. To achieve a meaningful comparison of the data obtained in the PFC and the S1 cortex, the authors need to determine whether the record from similar pyramidal cell populations.

We apologize for having not been precise about the specific location and type of pyramidal neurons in the original manuscript. Extracellular stimulation in PFC and S1 was always performed in layer 2, where the first large cell bodies, relative to the pia mater, are located within a cortical column. Therefore, we assume that the same cell populations were stimulated in both brain regions (van Aerde & Feldmeyer, Cereb. Cortex 2015; Oberlaender et al., Cereb. Cortex 2012; Lefort et al, Neuron 2009). To stick with the standard terminology, we refer to it in the manuscript as upper layer 2/3. We kept stimulation intensity as low as possible to ensure that only a few presynaptic cells within the target region were activated.

In S1, paired recordings were obtained from pyramidal neurons in layer 5A following the procedures and criteria described in detail in our previous work (Bornschein et al., Cell Rep. 2019; Bornschein et al., Front. Syn. Neurosci. 2019; Bornschein et al., Science 2025). Briefly, these criteria are as follows: close proximity to layer 4 and the barrels as well as the PPR of 0.78 (0.69-0.90), which is consistent with depression dominating in L5A (Frick et al., Cereb. Cortex 2008; Bornschein et al., Front. Syn. Neurosci. 2019) and different from L5B with PPR ≥ 1 (Lefort & Petersen, Cereb. Cortex 2017). Within layer 5A we did not attempt to further differentiate between pyramidal neuron types. For recordings in S1 with extracellular stimulation we focused on the same locations as for the paired recordings. We extended the corresponding section in Materials and Methods of the revised manuscript.

We agree that there is no clear layer 4 in PFC, making the distinction between layer 2/3 and layer 5 less clear. Layer 2/3 and layer 5 have approximately the same diameter (van Aerde & Feldmeyer, Cereb. Cortex 2015). Based on this, we performed recordings in upper layer 5 of PFC. We did neither morphologically nor electrophysiologically differentiate between pyramidal neuron cell types. It should be noted that within a cortical area (S1 or PFC), we did not find a difference between glutamatergic synapses from L2/3 onto L5PNs and L5PN-to-L5PN synapses, neither with regard to the EGTA-sensitivity of release nor with regard to the release probability. In particular, we found homogeneous results and similar variability in both, the examined connections in the PFC and in S1, with no discernible clustering in the data that would suggest stimulation of different cell populations. These findings suggest that excitatory inputs to L5PNs exhibit similar properties (PPR, *p*_N_, CD) irrespective of whether they originate in L2/3 or in neighbouring PNs in L5A. However, we do see significant differences between synapses in the different cortical areas S1 and PFC. Thus, intra-area specific differences in morphology and spiking patterns among pyramidal neurons appear to not be reflected on the level of their synapses.

The Reviewer probably refers to such differences and heterogeneity in morphology and spiking patterns of pyramidal neurons. If he/she has more specific differences in mind, it would be helpful if references for the significant heterogeneity could be given.

Please also note that the type of experiments we perform with paired recordings and long-lasting patch-clamp measurements is not suitable for analyzing population differences among pyramidal neuron types.

We refer to the problem of pyramidal neuron heterogeneity in the revised manuscript in the discussion.

“Patch-clamp recordings from L5PNs located in the upper layer 5 (L5A in S1) were established according to the criteria described in detail in our previous work on this connection in S1 (Bornschein et al., 2019b; Bornschein et al., 2025). Presynaptic neurons were stimulated extracellularly in upper layer 2/3 (L2/3-L5PN connections) straight above the patched L5PN or in on-cell mode in L5A right next to the postsynaptic cell (L5PN-L5PN connections; Figure 1).“

“We did neither morphologically nor based on spiking patterns differentiate further between PN subtypes within a given layer. However, within a cortical area (S1 or PFC) we did not find a difference between glutamatergic synapses from L2/3 onto L5PNs and L5PN to L5PN synapses, neither with regard to the EGTA sensitivity of release nor with regard to *p*_N_. In particular, we found homogeneous results and similar variability in both, the examined connections in the PFC and in S1, with no discernible clustering in the data that would indicate stimulation of different cell populations. These findings suggest that excitatory inputs to L5PNs exhibit similar properties (PPR, *p*_N_, CD) irrespective of whether they originate in L2/3 or in neighboring PNs in L5A. However, we do see significant differences between synapses in the different cortical areas S1 and PFC. Thus, intra-area specific differences in morphology and spiking patterns among PNs appear to be not reflected on the level of their synapses.“

(4) For the S1 cortex, in rats it has been found that L5 synaptic connection between pairs of L5a pyramidal cells and pairs of L5b pyramidal cells differ markedly with respect to mean EPSP amplitude, latency and coefficient of variation (cv, a surrogate measure for the synaptic release probability) (cf. Markram et al., 1997; Frick et al., 2008). It is therefore likely that PFC and S1 pre- and postsynaptic pyramidal cells are not only morphologically and electrophysiological distinct but also with respect to their synaptic properties. At least, the authors need to discuss these confounding issues and preferentially address them experimentally. For example, it would be helpful to demonstrate that paired recordings were made from the same pyramidal cell types, perhaps by documenting their morphology and/or firing patterns. In addition, they should discuss the marked difference in EPSP amplitude and putative release probability between their data and the earlier studies.

We agree that Markram et al. (J. Physiol. 1997) and Frick et al. (Cereb. Cortex 2008) provided highly valuable insights into synaptic transmission between pyramidal neurons in S1. We referred to their work in detail in our previous work on developmental changes in the presynaptic organization of transmitter release in L5APN synapses in S1 (Bornschein et al., Cell Rep. 2019). Both studies were performed in young rats and EPSPs were measured, whereas we worked in mice and recorded EPSCs. This impedes a direct comparison of amplitudes.

Markram et al. (J. Physiol. 1997) recorded in 2-week-old rats from thick tufted PNs, corresponding to L5BPNs. Given the longer lifespan and slower development of rats compared to mice, this likely reflects a maturation state that corresponds better to our previous measurements in 8 to 10-day-old mice. Markram et al. found small failure rate (median 7%), which is similar to what we found in our previous study for young L5APN synapses (low failure rates and high *p*_N_; Bornschein et al., Cell Rep. 2019).

The study by Frick et al. (Cereb. Cortex 2008) is closer to our present study and to the mature age window in our previous study, although they also recorded from rats but from L5APN-L5APN pairs in almost 3-week-old animals in S1. Again, EPSPs rather than EPSCs were recorded, impeding a direct comparison of amplitudes.

Both studies concluded, based on the synaptic failure rate and CV analysis of EPSP amplitude, that the synapses they investigated operate with high release probability. This is fully in line with our findings. Of note, we found no significant difference between L2/3-L5PN and L5PN-L5PN synapses within a given area, indicating that varibality on the synaptic level between PNs of a given area is not pronounced.

In order to further substantiate this, we determined the relative variability in median EPSC amplitudes to test whether there is a higher variability of recorded cell types in PFC compared to S1. The relative MAD (median absolute deviation) of EPSC amplitudes was 0.46 in PFC and 0.50 in S1. The similarity in these values argues against higher cell-type variability in PFC compared to S1. We have discussed the results of these studies in relation to our own findings.

“Two other previous studies on L5APN (Frick et al., 2008) and L5BPN (Markram et al., 1997) connections concluded that these synapses operate with high release probability, which nicely agrees with our previous (Bornschein et al., 2019b) and current results. It is remarkable that we did not even detect any differences between the L2/3-L5PN and L5PN-L5PN synapses within a given cortical area. Overall these results from different studies (Markram et al., 1997; Reyes and Sakmann, 1999; Frick et al., 2008; Bornschein et al., 2019b; Bornschein et al., 2019a) may indicate that variability on the synaptic level between PNs of a given area is not pronounced. In order to further substantiate this, we determined the relative variability in median EPSC amplitudes to test whether there is a higher variability of recorded cell types in PFC compared to S1. The relative MAD (median absolute deviation) of EPSC amplitudes was 0.46 in PFC and 0.50 in S1. The similarity in these values argues against higher cell-type variability in PFC compared to S1.

(5) In order to perform multiple probability fluctuation analysis (MPFA), a parabolic fit with a mere three points is inadequate, particularly because 2 mM and 5 mM Ca^2+^ are close to the peak of the variance-to-mean parabola, and only 1 mM Ca^2+^ is on its initial linear part. A more meaningful result would have been obtained with an additional Ca^2+^ concentration between 1.0 and 2.0 mM, as these are closer to the physiological range. In this context, the authors should have quoted the more recent and more detailed paper by the Silver group (Saviane and Silver, 2006; Lanore and Silver, 2016) and not just the Clements and Silver review paper.

We used only three Ca^2+^ concentrations for MPFA as these resulted in a low (<0.5), a medium (~0.5) and a large (>0.5) *p*_N_ condition, thereby clearly determining a parabola. Also Saviane and Silver (Nature 2006) performed MPFA with three extracellular Ca^2+^ concentrations (1, 2, and 8 mM). We have now cited this paper, as well as the more recent work by Lanore and Silver (Neuromethods 2016), in relation to the MPFA method. To verify the reliability of MPFA, the determined parameters were compared with values estimated based on the EPSC amplitudes (EPSC = *N p*_N_ *q*; PFC, 6 pA; S1, 48 pA) and failure rates (F = (1-*p*_N_)^*N*; PFC, 0.25, S1, 0.0001). The estimated values did in fact match those of the MPFA EPSCs in PFC: 8 pA, 5-15 pA, and S1: 29 pA, 18-53 pA; failure rates in PFC: 0.16, 0.08-0.28, and S1: 0, 0-0.03; see original manuscript.

To further support this, we have now conducted additional experiments using four Ca^2+^ concentrations. The results are consistent with those from the experiments using three concentrations. The additional Ca^2+^ concentration of 1.5 mM did not improve the parabolic fit, as it yielded *p*_N_ values very close to those determined with 2 mM Ca^2+^. Therefore, the additional experiments are shown in Author response image 1. The novel *p*_N_ data are included in the summary of *p*_N_ values (now n=6) in the results section and in Figure 3F.

**Author response image 1.** MPFA with four different extracellular Ca^2+^ concentrations. (A) MPFA of EPSC amplitudes recorded at the indicated [Ca^2+^]_e_ from L2/3-L5PNs in PFC. Top: Individual EPSCs (grey, average in black) recorded from L5PNs after extracellular stimulation in L2/3. Middle: Plot of EPSC amplitudes over time. Bottom: Corresponding mean-variance plot fitted with a parabola estimating the quantal parameters of release. *p*_N_ is for 2 mM [Ca^2+^]_e_. Recordings were made in the presence of 10 µM Bicuculline, 0.25 mM Kynurenic acid and 50 µM 2-Amino-5-phosphonovaleriansäure. (B) As in (A), but for a L2/3-L5PN connection in S1. (C) Summary of determined *p*_N_ values in PFC and S1 (dots represent individual experiments; P=0.485, Mann-Whitney U rank-sum test).

Additionally, we performed bootstrap analyses with 10,000 replicates which were generated with replacement from the original data sample. Distributions of bootstrap 25% trimmed means showed a clear separation for *N* and *q* between PFC and S1 but not for *p*_N_. The bootstrap results are shown in Author response image 2.

**Author response image 2.** Bootstrap analysis (A-C) Distribution of bootstrap 25% trimmed means of the quantal parameters *p*_N_ (A), *N* (B) and *q* (C) in PFC (orange), S1 (blue) and S1 with gDGG (light blue). 10,000 bootstrap replicates were generated with replacement from the original data sample obtained by MPFA in L5PN-L5PN connections (cf. Figure 3D). (D) Same as in (A) but for MPFA in L2/3-L5PN connections.

(6) Methods: The authors should clarify whether their paired recordings from L5 pyramidal cells involved whole-cell recordings from both pre- and postsynaptic neurons. From Figure 1B, it appears as if the presynaptic neurons were not recorded in whole cell mode but rather stimulated in cell-attached mode. This is also reflected in the artefact visible in the current trace recorded in the postsynaptic neuron. The authors should explicitly state their methodological approach and mention how reliable the timing of the presynaptic action potential was under these circumstances. The same holds true for the extracellular stimulation protocol. A significantly more detailed description of the experimental protocol is necessary here.

In the paired recordings, presynaptic cells were stimulated in the cell-attached mode. For presynaptic EGTA application the whole-cell configuration was established after re-patching to allow buffer perfusion of the presynaptic L5PN. This is described in the methods section of the original version of the manuscript. We extended this description as follows:

“In paired recordings, presynaptic L5PNs were stimulated in on-cell configuration (200-500 mV, 1-2 ms). In the chelator wash-in experiments, presynaptic neurons were repatched with a pipette solution supplemented with 10 mM EGTA (K-gluconate concentration was reduced to 135 mM to adjust osmolarity) and whole-cell configuration was established to allow EGTA perfusion of the presynaptic neuron.”

The amplitudes were determined by fitting a product of two exponential functions to the baseline-subtracted currents, which allows for independent adjustment of the time constants of the rising and falling phases and minimizes noise effects (cf. Bornschein et al., J. Physiol. 2013). Synaptic delays were determined from the onset of stimulation to the fitted EPSC onset. We added this more detailed explanation to the methods section. in the timing of the presynaptic action potential was similar in recordings from PFC and S1. This applies to the paired-recordings with on-cell stimulation of presynaptic neurons as well as to the extracellular stimulation experiments.

“Synaptic responses were determined by fitting a product of two exponential functions to the baseline-subtracted currents, which allows for independent adjustment of the time constants of the rising and falling phases and minimizes noise effects (Bornschein et al., 2013). Synaptic delays were determined from the onset of stimulation to the fitted onset of the EPSC. PPRs were calculated by dividing the second amplitude of two consecutive EPSCs by the first.”

(7) Methods: The authors use Student's t-test for data comparison. The authors should verify that the data distribution was indeed normal, e.g. by using a Shapiro-Wilk test. If this is not the case, non-parametric tests should be used.

We typically used non-parametric tests as stated in the figure legends of the corresponding figures. We have now explained the abbreviations for the Mann-Whitney U test (MWU) and the Wilcoxon signed-rank test (WSR) in the figure legends. A paired t-test was used only in Figure 5F after testing for normal distribution with the Shapiro-Wilk test. This is described in the methods section of the original version of the manuscript. Additionally, results of the Shapiro-Wilk test were now included in the figure legends.

“Normality was tested using the Shapiro-Wilk test. Normally distributed data were compared with the t-test (two groups) or a one-way ANOVA (more than two groups). Non-normally distributed or small samples of data were compared with the Mann-Whitney U rank-sum test (MWU; two groups) or a Kruskal-Wallis ANOVA on ranks (more than two groups). (…). To compare pre- and post-treatment data the paired t-test or the Wilcoxon signed-rank test (WSR) was used, depending on the distribution of the data.”

**Reviewer #3 (Public review):**

Summary:

In this manuscript, Max Schwarze and colleagues examined the coupling distance between presynaptic Ca^2+^ channels and the vesicular release sensor at neocortical synapses in mice. They propose that Ca^2+^ channel-release sensor coupling differs across cortical areas, with relatively loose (microdomain) coupling in prefrontal cortex (PFC) and tighter (nanodomain) coupling in primary somatosensory cortex (S1) for comparable pyramidal-neuron synapse types. To test this, they combine paired recordings and minimal stimulation with chelator manipulations (EGTA/BAPTA), mean-variance/MPFA-style analyses, presynaptic Ca^2+^ imaging, and computational modeling. They conclude that presynaptic coupling organization is area-specific in the mature cortex and contributes to regional differences in synaptic timing, reliability, and short-term plasticity.

Strengths:

This study tackles an important question and is strengthened by a cohesive body of evidence assembled from multiple complementary approaches. A major asset is the inclusion of high-value datasets, particularly the paired recordings between L5 pyramidal neurons and the systematic assessment of EGTA sensitivity, which provide a solid functional foundation for the authors' central claims. The work is further distinguished by its genuinely multimodal design: combining electrophysiology with presynaptic calcium imaging (and integrating these observations with quantitative analyses and modeling) offers a more mechanistic view of neurotransmitter release than any single method could provide. Overall, the direct, within-framework comparison of presynaptic release-control mechanisms across cortical areas for comparable synapse types is compelling and gives the conclusions a level of robustness and interpretability that is often difficult to achieve in studies of cortical synaptic diversity.

Weaknesses:

Several aspects would benefit from clearer explanation, stronger integration with the existing literature, and a more explicit discussion of limitations and potential confounds. Without these additions, some conclusions remain speculative. Throughout the manuscript, the authors also often imply that different measurements reflect the same underlying synapse population. This is unlikely to be strictly true across all experiments and makes it difficult to integrate results from the various approaches into a single, unified set of functional synaptic properties. In addition, some statements-particularly those linking coupling mode to "higher-order neocortical functions"-appear broader than what is directly supported by the experiments and should be tempered or more precisely scoped.

Below, I list several topics that could help better frame the main findings of the present study and clarify how it relates to previously published work.

We would like to thank the reviewer for the comprehensive and detailed assessment of our manuscript and his/her overall positive evaluation. We have addressed all of the reviewer's points. We expanded the model description and discussion, and slightly toned down our conclusion.

(1) The authors use EGTA sensitivity of EPSCs (together with additional metrics) to argue that S1 and PFC synapses differ in Ca^2+^ channel-release sensor coupling. While this is a plausible interpretation, EGTA effects are not uniquely determined by coupling distance and can also reflect differences in Ca^2+^ entry kinetics, action potential waveform, endogenous buffering/extrusion, or release-sensor/vesicle state. The authors use a constrained modeling approach, but the rationale for the different constraint sets is not fully clear from the current description. It would be helpful to expand and clarify the Methods section to explain how these constraints were defined, justified, and applied (and how alternative constraint choices would affect the results). In this context, the Abstract's broader claim that the study "reveals microdomain coupling as a presynaptic structure-function correlate of higher-order neocortical functions" appears overstated. Given the well-known diversity of cortical synapses even within a single region (e.g., synapses onto different interneuron subclasses or different PN cell types, extracortical sources like thalamus), the authors should clarify the intended scope: is the conclusion meant to apply broadly across synapse classes in S1 and PFC, or only to the specific connection type(s) examined here?

We would like to thank the reviewer from pointing out that our description fell a bit short, in particular with respect to the interpretation of the EGTA effects. We addressed the points as follows in the revised manuscript: We discussed the interpretation of EGTA effects in more detail. We toned down the concluding statement in the last sentence of the Abstract.

“Differences in the sensitivity of release to low to moderate concentrations of EGTA (≤ 30 mM) are a standard indicator of differences in the coupling distance (e.g. Adler et al., 1991; Bucurenciu et al., 2008; reviewed in Eggermann et al., 2012; Vyleta and Jonas, 2014; Kusch et al., 2018; Bornschein et al., 2019b). *p*_N_ is determined by the size of the Ca^2+^ signal at the release sensor and the binding kinetics and affinity of the sensor. The former in turn is determined by the details of the Ca^2+^ influx and the diffusional coupling distance between the VGCCs and the sensor. The similarity of Ca^2+^ signals between synapses in PFC and S1 (Figure 4) indicates that Ca^2+^ influx is similar between boutons, although more subtle differences in the influx kinetics may have remained undetected in these volume-averaged signals. Regarding sensor affinity, results in a previous study indicate that differences in EGTA sensitivity show differences in coupling rather than sensor affinity even if *k*_on_ of the sensor and its affinity should differ as much as ten-fold, which appears to be an unlikely scenario given that even the two major isoforms of Synaptotagmin that trigger synchronous release differ by less than a factor of three to four in their affinity (Bollmann et al., 2000; Schneggenburger and Neher, 2000; Bornschein et al., 2025). Finally, the increase in the PPR induced by the application of Cd^2+^ further supports our conclusion of microdomain coupling in the PFC synapses (Scimemi and Diamond, 2012).”

“They suggest that microdomain coupling in pyramidal neuron synapses could be a presynaptic structure-function correlate of higher order neocortical functions.”

(2) The chelator logic is sound in principle, but the Discussion should more explicitly acknowledge standard caveats and alternative explanations. The authors partly address this by including presynaptic Ca^2+^ imaging and modeling, yet it would help to explain more clearly how the combination of (i) chelator sensitivity, (ii) presynaptic Ca^2+^ signals, and (iii) model constraints rules out-or substantially reduces the likelihood of-changes in AP waveform, Ca^2+^ influx kinetics, buffering/extrusion, or sensor/vesicle state as the primary drivers. In addition, recent hypotheses emphasizing vesicle priming and/or release-site occupancy as contributors to apparent EGTA sensitivity should be discussed as a complementary or alternative interpretation.

Please see above the first part of the discussion to point one.

(3) A substantial portion of the S1 comparison appears to rely on previously published datasets. This should be made unambiguous in the Results and Methods, and it would be helpful to summarize this clearly (e.g., in a table indicating which figures/analyses use new data versus reanalysis of published data). If this information is already present, it should be highlighted more prominently.

Please excuse us for not having made it clearer which data had already been published. Only the paired recordings from L5PN-L5PN connections in S1 were obtained in previous studies and partially reanalyzed. Paired recordings on the same synapses in PFC as well as all recordings from L2/3-L5PN connections in PFC and S1 were obtained in the present study. At your suggestion, we have added Table 1 highlighting which data and associated figures are from this study and which were acquired in previous studies (Bornschein et al., Cell Rep. 2019; Bornschein et al., Front. Syn. Neurosci. 2019).

(4) The modeling is informative, but the choice of a specific VGCC-release-site geometry and channel arrangement is not sufficiently justified. The manuscript adopts a particular spatial configuration, yet the rationale for selecting this geometry, rather than other plausible architectures discussed in the literature, is not clearly explained, nor is it meaningfully revisited in the Discussion. The authors should justify why the same organization is assumed across two distinct cortical areas and, ideally, include (or at a minimum discuss) a sensitivity analysis showing how key inferences (e.g., coupling distance and channel number) depend on the assumed geometry.

We extended the discussion of why a ring-like structure of VGCCs was assumed in the model.

“The microdomain was assumed to be formed by a ring-like structure of VGCCs around a vesicle (Figure 5D). This topography was chosen because such a microdomain was found to best predict the experimental data of transmitter release from PNs in young S1 (Bornschein et al., 2019b). Other previously described distributions of VGCCs suitable to reproduce release data cover random distributions of VGCCs (Scimemi and Diamond, 2012), VGCC clusters (Meinrenken et al., 2002; Nakamura et al., 2015), and exclusion zones (Keller et al., 2015). In the early S1, all of these models predicted a higher EGTA sensitivity of the microdomain, however, these models provided a poorer fit to the full set of the experimental data than the ring-like structure (Bornschein et al., 2019b). Since the experimental data from PNs in the mature PFC were similar to those in young S1, these other microdomain models were not tested explicitly here.”

(5) The calcium imaging data are valuable, but given the diversity of synapses within each cortical layer, it is not clear that imaged boutons can be confidently assigned to the specific connection types being interrogated electrophysiologically. A substantial fraction of boutons likely corresponds to different postsynaptic targets (including interneurons and distinct pyramidal-cell classes), and this heterogeneity could complicate interpretation. This limitation should be discussed explicitly

Excitatory pyramidal cells make up 80-85% of cortical neurons, with the highest density in layer 5 (Keller et al., Front. Neuroanat. 2018). In the somatosensory cortex, inhibitory synapses account for only about 10% (Santuy et al., Brain Struct. Funct. 2018). We imaged a large number of presynaptic boutons within layer 5 (about 10 boutons per cell, in total 85 boutons in PFC and 100 boutons in S1, numbers of boutons were now included in Figure 4). In this respect, the impact of inhibitory synapses is minor. Since connectivity between neighboring PNs in layer 5A is high (Feldmeyer, Front. Neuroanat. 2012), we assume that a large proportion of the imaged boutons target neighbouring L5PNs. We added a sentence on potential postsynaptic targets in the results section.

“The imaged presynaptic boutons most likely connect to neighboring pyramidal cells, as connectivity between L5PNs in layer 5A is high (Feldmeyer, 2012). Nevertheless, a small proportion of other postsynaptic targets, such as interneurons, cannot be ruled out.”

(6) In unitary connections, the authors assess EGTA effects alongside other functional parameters (strength, delay, short-term plasticity), which is a major strength. However, for L2/3 to L5 connections, it appears that EGTA sensitivity was tested primarily using extracellular stimulation. Given anatomical and circuit differences between PFC and S1, extracellular stimulation may recruit different synapse populations across regions, potentially confounding regional comparisons of EGTA sensitivity. This limitation should be acknowledged explicitly. While I am not requesting technically demanding L2/3↔L5 paired recordings in S1, the possibility that different synapse identities are being sampled should be treated as a meaningful source of uncertainty. The Discussion would also benefit from placing the magnitude of EGTA effects in the context of prior "loose coupling" literature, where comparatively large EGTA effects have been reported in some systems. In addition, the reported difference between adult PFC EGTA effects and S1 inhibition appears small (on the order of <10%) and should be interpreted cautiously, especially given that PFC and S1 mature on different timelines and P21-P26 is unlikely to reflect a mature PFC circuit state. The adult cohort (P90-P100) is therefore important, but the age mismatch complicates PFC-S1 comparisons; ideally, S1 should be assessed at matched ages, or this limitation should be discussed explicitly. Finally, for statistical robustness, in panel D of Figure 2, were the comparisons corrected for multiple testing to control Type I error?

EGTA sensitivity was examined in PFC and in S1, for two connections in each region - using paired recordings for L5PN-L5PN connections and using extracellular stimulation for L2/3-L5PN connections. The L5PN-L5PN data from S1 were collected in an earlier study (Bornschein et al., Cell Rep. 2019; Bornschein et al., Front. Syn. Neurosci. 2019), have now been reanalyzed for the test period between 20 and 30 min, and included in Figure 2B for the sake of consistency (see Table 1). To emphasize this point, despite stimulating different input synapses with different stimulation methods, we obtained similar results in the respective brain regions. This suggests that the EGTA sensitivity observed in the investigated PFC connections is not a solely synapse-specific property.

It is difficult to compare the absolute EGTA sensitivities from different synapses from different publications, since EGTA effects do not depend exclusively on the coupling distance, as the reviewer also noted in point 1. They are, among other factors, influenced by the Ca^2+^ sensitivity of the release machinery, which differs between our Syt1-expressing cortical synapses and Syt2-expressing synapses in other brain regions (Schneggenburger et al., Nature, 2000; Bollmann et al., Science, 2000; Bornschein et al., Science 2025), such as the calyx of Held or the cerebellar basket to Purkinje cell synapse. Furthermore, direct patching and loading of the presynaptic bouton with EGTA - as feasible at the calyx of Held and other large synapses - results in higher effective EGTA concentrations compared to somatic loading of presynaptic terminals, despite identical pipette concentrations. The buffer-AM method introduces additional uncertainty regarding the effective intra-bouton EGTA concentration, since the loading efficacy has to be estimated. Thus, although differences in EGTA sensitivity primarily show differences in coupling distances, the comparison of absolute values between different publications is difficult. Consistently, data-constrained models are used to estimate the coupling topography and to compare these topographies rather than comparing the absolute EGTA effects (e.g. Buccurenciu et al., Neuron, 2008; Vyleta and Jonas, Science, 2014; Bornschein et al., Cell Rep., 2019; Chen et al., Neuron, 2024; Bornschein et al., Science, 2025).

We include a note on this in the discussion.

“Thus, although the absolute EGTA sensitivity is influenced by different factors, which necessitates data-constrained models for quantitative comparisons, the general sensitivity of release to EGTA indicates loose coupling.”

In mouse neocortex postnatal maturation in S1 and PFC follows the same time course. Kroon et al. (Sci. Rep. 2019) reported that maturation of dendritic morphology and intrinsic properties of pyramidal neurons occurs within the first two weeks after birth, now cited in the discussion. Therefore, it is unlikely that the EGTA effect in PFC is due to a delayed maturation. The difference in EGTA sensitivity between PFC at P90-100 and S1 at P21-26 is indeed small but significant (P=0.009, Mann-Whitney-U rank sum test).

“Since postnatal development follows the same time-course in mouse PFC and S1 and occurs predominantly within the first two weeks after birth, (…) (Kroon et al., 2019).”

Thank you for the advice concerning statistical robustness. We replaced the Mann-Whitney-U rank sum test in Figure 2D by a one-way ANOVA and performed a Holm-Sidak post-hoc test correcting for multiple comparisons. Similarly, ANOVA was used to compare more than two groups in Figures 1K and 2F. We changed the corresponding P values and tests in the figure legends and added the performed post-hoc tests in the methods section.

“For multiple comparisons post-hoc testing was performed with the Holm-Sidak (one-way ANOVA) or Dunn´s method (ANOVA on ranks).”

(7) Alterations in initial release probability are often associated with changes in short-term plasticity. In the present manuscript, the authors report similar initial release probability at PFC and S1 synapses, yet observe differences in short-term plasticity profiles. The mechanistic basis for this apparent dissociation is not addressed and should be discussed explicitly, including potential explanations.

Various other factors besides *p*_N_ can influence short-term plasticity, that are the coupling distance, the number of occupied release sites (*N*_occ_), the replenishment of *N*_occ_ or the recruitment of newly formed *N*_occ_ as well as the expression of endogenous Ca^2+^ buffers (Blatow et al., Neuron 2003; Felmy et al., Neuron 2003; Matveev et al., Biophys. J. 2004; Neher, Cell Calcium 1998; Regehr, CSH Perp. Biol. 2012) or fascilitation sensors (Turecek & Regehr, J. Neurosci. 2018; Shin et al., eLife 2025). Traditionally, *p*_N_ had been assumed to have a major impact on short-term plasticity (STP) which is indeed the case at low replenishment rates (e.g. Feldmeyer and Radnikow, J. Physiol. 2009; Zucker and Regehr, Ann. Rev. Physiol. 2002). But at several synapses very fast replenishment rates have been described driving a progressive overfilling of the initial RRP and increasing *N*_occ_ above baseline levels (Brachtendorf et al., Front. Cell. Neurosci. 2015; Doussau et al., eLife 2017; Miki et al., Neuron 2016; Valera et al., J. Neurosci. 2012) making replenishment the stronger determinant of STP.

Additionally, the size and organization of sub-pools from which vesicle recruitment and release occurs affects the speed and reliability of vesicular release. In our previous study on L5PN-L5PN connections in S1 we found that developmental tightening of CDs was associated with an increase in PPR without altering *p*_N_ (Bornschein et al., Cell Rep. 2019). We could show that the maturation of a replenishment pool during postnatal development increases vesicle recruitment and reliability thereby affecting STP (Bornschein et al., Front. Syn. Neurosci. 2019).

We discussed this in the revised manuscript.

“Classically, *p*_N_ was considered as the major determinant of short-term plasticity (e.g. reviewed in Zucker and Regehr, 2002; Feldmeyer and Radnikow, 2009). More recently other factors, including the number of occupied release sites, their replenishment or an increase in their occupancy, or the expression of endogenous Ca^2+^ buffers have been considered as more important determinants of short-term plasticity (Rozov et al., 2001; Blatow et al., 2003; Felmy et al., 2003; Matveev et al., 2004; Bornschein et al., 2013; Miki et al., 2016; Doussau et al., 2017; Jackman and Regehr, 2017; Neher and Brose, 2018). (…)”

Short-term plasticity changes during postnatal development at different cortical PN connections without alterations in *p*_N_ (Reyes and Sakmann, 1999; Bornschein et al., 2019a). For L5PN-to-L5PN connections these differences were found to result from the maturation of an intermediate replenishment vesicle pool (Bornschein et al., 2019).

(8) There are multiple instances where the text appears to cite non-existent or misnumbered figure panels (e.g., references to "Figure 4G-I / 4J" when the relevant material appears elsewhere). These should be corrected throughout, as they currently reduce readability and confidence.

We apologize for the misnumbering which originated from a previous version of this manuscript. Figure 4G-J is actually Figure 5A-D. We corrected the references to Figure 5 in the methods section.

(9) The Methods describe P21-P26 animals, whereas the Results include older cohorts (e.g., P90-P100) and additional regions (e.g., mPFC). The Methods should be updated so that all cohorts and regions analyzed in the Results are fully described.

Thank you for thoroughly reading the methods. We added the missing cohort (P90-100) and brain region (mPFC) to the method section.

“C57BL/6J mice at P21-26 and P90-100 of either sex were decapitated under deep Isoflurane (Curamed) inhalation anaesthesia. (…) Coronal neocortical slices (150-250 μm thick) were cut from the lateral PFC, medial PFC (mPFC) or S1 region (Figure 1A) with a vibratome (HM 650 V, Microm).”

**Recommendations for the authors:**

**Reviewer #1 (Recommendations for the authors):**

These are mostly points for discussion; there is no need for additional experiments.

(1) Discuss potential effects of (re-)patching on presynaptic physiology and the "control" time course in Figure 2A.

The repatching strategy is a strength, but it also introduces opportunities for physiological drift (dialysis effects, changes in access resistance, altered excitability, and the switch to presynaptic stimulation in whole-cell after repatching). Please discuss (and, if already quantified, briefly report) why EPSC amplitudes appear to increase in the control time course after repatching (as seen in Figure 2A). Even a short explanation (e.g., run-up after whole-cell access, recovery from on-cell stimulation, washout of endogenous buffering, improved spike waveform reliability, etc.) plus reassurance that baseline stationarity criteria were met would strengthen confidence in the repatching-based inference.

Baseline recordings were usually performed with the presynaptic neuron in cell-attached mode. In this configuration intracellular ion concentration, second messenger systems as well as cell-specific resting membrane potential stay essentially unaffected. When switching to whole-cell mode after re-patching of the presynaptic cell, the pipette solution determines the intracellular environment, which may also affect second messenger systems and mobile endogenous buffers will be washed out. Although layer 5 pyramidal neurons do not express relevant concentrations of these mobile buffers (Helmchen et al., Biophys. J., 1996; Tran and Stricker, Biophys. J., 2018; Bornschein et al., Cell Rep., 2019), this might have contributed to the moderate and temporary run-up after whole-cell access to presynaptic cells.

In postsynaptic neurons, we also routinely controlled the stability of R_s_ and I_leak_ during our prolonged measurements. In the controls, we found a median initial increase in relative EPSC amplitudes to 1.17 (1.02-1.38) between 0 and 10 min after the presynaptic whole-cell access was established, which correlated with a temporary decline in R_s_ in 3 out of 6 recordings. EPSC amplitudes returned to their baseline values after 20 min at the latest (1.02, 0.75-1.20). We added potential reasons for the temporary amplitude increase in the controls in the results section.

“In control recordings a temporary initial increase in EPSC amplitudes after whole-cell access to the presynaptic neuron was evident. Although L5PNs do not express large concentrations of mobile buffers (Helmchen et al., 1996; Tran and Stricker, 2018; Bornschein et al., 2019b), their wash-out might have contributed to the temporary run-up. On the other hand, run-up correlated with a temporary decline in R_s_ in some recordings (3 out of 6). Run-up effects normalized after 20 min at the latest (1.02, 0.75-1.20).”

(2) Clarify interpretation/robustness of MPFA-derived "N" given the large range, and discuss uncertainty from using only three [Ca^2+^]e conditions for L2/3-L5PN MPFA.

The manuscript reports that "N" is markedly smaller in PFC than S1 (median ~2.1 vs 8), but the S1 range is very broad (3-19).

(a) Briefly discuss whether/why such a wide N range is expected and how it should be interpreted (binomial "N" vs anatomical release sites; sensitivity to CV assumptions; potential dependence on connection geometry, bouton number, dendritic filtering, etc.).

(b) Add a short statement on uncertainty/identifiability when fitting MPFA with only three conditions for L2/3-L5PN (e.g., whether confidence intervals/bootstraps were examined; how stable q and N are to small changes in the variance estimates). Even a qualitative note would help readers judge how much weight to put on the absolute N estimates versus the overall cross-area trend.

(a) In our previous study on this connection we determined a wide range for *N* (8, 3-19) even though we used four extracellular Ca^2+^ concentrations in MPFA (Bornschein et al., Cell Rep. 2019). The binomial parameter N can be considered to represent the number of release sites, including empty release sites (see Brachtendorf et al., Front. Cell. Neurosci. 2025). The range of release sites is likely to reflect the variability in the number of anatomical synaptic contacts ranging from 1 to 6 for these synapses (Frick et al., Cereb. Cortex 2008). Since 1 to 3 active zones/release sites per synaptic contact appear to be typical for small cortical synapses (e.g. Xu-Friedman et al., J. Neurosci. 2001), this results in a wide range of 1-18 release sites per connection.

In our previous study (Bornschein et al., Cell Rep. 2019), we also investigated the effects of different values of CV1 and CV2 by repeating the MPFA fitting procedures for different combinations of CV1 and CV2 ranging from 0.1 to 1 each. We have quantified a deviation of ≤10% in the estimates of vesicular release probability from the typically used CV values of 0.3 across a wide range of CV value combinations (see also Schmidt et al., Curr. Biol. 2013). We have added a note regarding CV sensitivity in the methods section.

“For CV assumptions that deviate from the standard value of 0.3, deviations in the calculated *p*_N_ values of less than 10% are to be expected (Schmidt et al., 2013; Bornschein et al., 2019b).”

(b) The three Ca^2+^ concentrations we used for MPFA resulted in a low (<0.5), a medium (~0.5) and a large (>0.5) *p*_N_ condition. With this, a parabola is uniquely determined by three parameters. To further ensure the reliability of the parameters determined by MPFA, we compared them to values estimated from EPSC amplitudes (EPSC = *N p*_N_ *q*; PFC, 6 pA; S1, 48 pA) and failure rates (F = (1-*p*_N_)^*N*; PFC, 0.25, S1, 0.0001), which yielded values similar to those from MPFA (EPSCs in PFC: 8 pA, 5-15 pA, and S1: 29 pA, 18-53 pA; failure rates in PFC: 0.16, 0.08-0.28, and S1: 0, 0-0.03; see original manuscript).

To further support this, we have now conducted additional experiments using four Ca^2+^ concentrations. The results are consistent with those from the experiments using three concentrations. The additional experiments are shown for review purposes in Figure R1. The novel *p*_N_ data are included in the summary of *p*_N_ values (now n=6) in the results section and in Figure 3F.

Additionally, we performed bootstrap analyses with 10,000 replicates which were generated with replacement from the original data sample. Distributions of bootstrap 25% trimmed means showed a clear separation for *N* and *q* between PFC and S1 but not for *p*_N_. The bootstrap results are shown for review purposes in Author response image 2 (cf. point 5 of Reviewer#2).

(3) Broaden the discussion, e.g. by linking to nanodomain/microdomain coupling as a general strategy for stimulus encoding, including sensory periphery examples.

The work will resonate beyond the cortex if the authors explicitly connect their findings to broader principles: how the spatial coupling regime shapes the transfer function between Ca^2+^ entry and vesicle fusion, thereby tuning reliability, timing, and dynamic range. Requested addition: Please consider adding a short subsection discussing analogous implementations in the sensory periphery, especially ribbon synapses of cochlear inner hair cells and rod photoreceptors, where nanodomain coupling has been discussed as a key determinant of encoding and release dynamics. Also, citing relevant work such as that by Scimemi and Diamond 2012 would further strengthen the paper.

We agree that the work by Scimemi and Diamond (J.Neurosci. 2012) is important and we cited and discussed their work in several of our previous publications. We now also included the paper in the revised version of the present manuscript. As requested, we also included a discussion on findings from ribbon type synapses and also from the neuromuscular junction.

“Nanodomain coupling was also found in the peripheral nervous system, in particular at retinal (Singer and Diamond, 2003; Jarsky et al., 2010) and auditory (Moser and Beutner, 2000; Brandt et al., 2005) ribbon-type synapses and at the neuromuscular junction (Harlow et al., 2001; Shahrezaei et al., 2006). These synapses have highly specialized properties and appear to be optimized for very reliable transmission and, in the case of ribbon synapses, also for high-frequency coding of sensory information (reviewed in Matthews and Fuchs, 2010; Eggermann et al., 2012). Thus, it appears that synapses in the sensory pathways, in particular those engaged in reliable high-frequency coding of sensory information, both in the periphery and in the lower processing stages of the CNS, up to primary sensory cortices, operate with nanodomain coupling. In the executing motor pathway, the neuromuscular junction uses nanodomain coupling and, as recent results from our group suggest, also PNs in the primary motor cortex (Yarim et al., in preparation). It is tempting to speculate that complete loops from or to the primary cortices to their peripheral target organs operate with nanodomains. Microdomain coupling, on the other hand, appears to come into play only if integration of information from multiple sources and plasticity are the main focus, as at certain synapses in PFC (this study) or hippocampus (Vyleta and Jonas, 2014).”

“The microdomain was assumed to be formed by a ring-like structure of VGCCs around a vesicle (Figure 5D). This topography was chosen because such a microdomain was found to best predict the experimental data of transmitter release from PNs in young S1 (Bornschein et al., 2019b). Other previously described distributions of VGCCs suitable to reproduce release data cover random distributions of VGCCs (Scimemi and Diamond, 2012), VGCC clusters (Meinrenken et al., 2002; Nakamura et al., 2015; Rebola et al., 2019), and exclusion zones (Keller et al., 2015; Rebola et al., 2019). In the early S1, all of these models predicted a higher EGTA sensitivity of the microdomain, however, these models provided a poorer fit to the full set of the experimental data than the ring-like structure (Bornschein et al., 2019b). Since the experimental data from PNs in the mature PFC were similar to those in young S1, these other microdomain models were not tested explicitly here.”

“(…) Finally, the increase in the PPR induced by the application of Cd^2+^ further supports our conclusion of microdomain coupling in the PFC synapses (Scimemi and Diamond, 2012).”

(4) Address limitations of basal/resting Ca^2+^ estimates and make explicit that measured Ca^2+^ signals are volume-averaged (not microdomain) readouts.

(a) The reported basal [Ca^2+^]i values are in the ~tens of nM range. Given the stated in vitro KD for Fluo-5F in the authors' pipette solution (439 nM), the resting estimates are far below KD; this does not invalidate the approach, but it does warrant a brief discussion of sensitivity/uncertainty (influence of Rmin estimation, background subtraction, and how errors propagate into basal [Ca^2+^]i). Repeating experiments is not necessary-just clearer framing of limitations.

(b) Please also emphasize more prominently (ideally in Results and/or Discussion) that the bouton signals are volume averaged and therefore do not directly report calcium microdomains at active zones or nanodomains at release sensors. The Methods already state this point; echoing it in the main text would prevent over-interpretation by readers.

(a) We agree that Fluo5F is less suitable for determining absolute basal calcium levels. In a previous study (Bornschein et al., Science 2025) we determined the basal Ca^2+^ concentration with OGB1 (K_D_=166 nM; basal [Ca^2+^]_i_=44 nM, 24-58 nM, n=43 boutons from 10 cells) and observed no significant difference to basal [Ca^2+^]_i_ values determined with Fluo5F despite the K_D_ of 439 nM (31 nM, 16-54 nM, 14 boutons from 3 cells; P=0.204, MWU; data not published). We added this limitation to the results section and swapped Figure panels 4E and F for confluence. The calibration curve of Fluo5F as well as the comparison to basal [Ca^2+^]_i_ values determined with OGB1have been included in Figure S4.

“The quantification of absolute basal [Ca^2+^]_i_ was limited by the *K*_D_ of Fluo5F (439 nM), which slightly underestimated basal [Ca^2+^]_i_ values in comparison to quantification with OGB1 (K_D_=166 nM, Figure S4). Nevertheless, relative comparison of basal [Ca^2+^]_i_ yielded no significant differences between PFC (30 nM, 21-34 nM) and S1 (22 nM, 13-38 nM; Figure 4F).”

(b) In the results section, we have now emphasized that volume-averaged Ca^2+^ signals were measured.

“We performed dual-dye two-photon Ca^2+^ imaging (Sabatini et al., 2002) to quantify volume-averaged Ca^2+^ signals at presumed presynaptic boutons located on axon collaterals of L5PNs in PFC and in S1.”

**Reviewer #2 (Recommendations for the authors):**

(1) For a meaningful comparison, recordings from the PFC and the S1 cortex of the same animals should be undertaken. Additionally, I suggest performing additional experiments regarding the different cell types of L5 pyramidal cells in layer 5a.

We performed new experiments to determine EGTA sensitivity in PFC and S1 from the same animal. The results from these experiments agree with the previous results. They are included in the results section , in Figure 2C-F and in Figure S3A-C.

Additionally, we extended the discussion on the examined cell types. For S1 cortex we refer in more detail to our previous work, where we described in depth where and under consideration of which criteria our recordings were established and that based on these criteria we recorded from pyramidal neurons in layer 5A in S1 (Bornschein et al., Cell Rep. 2019; Bornschein et al. Front. Synapt. Neurosci. 2019; Bornschein et al., Science 2025). Within layer 5A, we did not attempt to further distinguish between types of pyramidal neurons. We include this in the methods section.

“Patch-clamp recordings from L5PNs located in the upper layer 5 (L5A in S1) were established according to the criteria described in detail in our previous work on this connection in S1 (Bornschein et al., 2019b; Bornschein et al., 2025). Presynaptic neurons were stimulated extracellularly in upper layer 2/3 (L2/3-L5PN connections) straight above the patched L5PN or in on-cell mode in L5A right next to the postsynaptic cell (L5PN-L5PN connections; Figure 1).”

For the recordings in PFC and heterogeneity in pyramidal neuron types we refer to our detailed response to the point 3 of Reviewer 2. There we also discuss that the heterogeneity in morphology and spiking patterns is probably not reflected on the synaptic level. We would also like to emphasize that the type of experiments we perform with paired recordings and long-lasting patch-clamp measurements is not suitable to differentiate between subpopulations of pyramidal neurons. This would require successful recordings form several tens of different pyramidal neurons, which is not feasible in our type of experiment. We discuss this limitation of the discussion.

(2) The authors need to comment in depth on their MPFA data, and if feasibl,e perform additional experiments.

Concerning the robustness of quantification of synaptic parameters by MPFA, we refer to our comments on point 2b of the recommendations for the authors to Reviewer 1. Additionally, we performed new MPFA experiments with four extracellular Ca^2+^ concentrations that agree with our results with three Ca^2+^ concentrations.

(3) The statistical analysis should be revised and a test for the normality of data distribution should be implemented.

A test for normal distribution (Shapiro-Wilk test) has already been described in the methods section in the previous version of this manuscript.

(3) Figure 1A is somewhat misleading because it could suggest that the authors have performed dual recordings in identified PFC pyramidal cells.

We added “L2/3 or L5” to the stimulation panel of Figure 1A to illustrate that we stimulated either extracellularly in L2/3 or L5PNs directly via the patch pipette.

(4) Is the relative variance of the mean EPSC amplitude and latency between connections larger in the PFC connections than in S1 cortex? This could indicate a variability in cell types.

The relative variance of EPSC amplitudes calculated as median absolute deviation (MAD) was 0.46 in PFC and 0.50 in S1 arguing against differences in the variability in cell types. The larger variability in delays expressed as SD_Delay_ is the result of the larger coupling distance in PFC compared to S1 (Bullmann et al., J. Neurosci. 2024). Consequently, also the relative MAD is larger (0.89) in PFC compared to S1 (0.14) and is therefore not able to detect differences in the variability of recorded cell types.

(5) Reyes and Sakmann (1999) have previously described differences for L2/3-L5b and L5b-L5b synaptic connections in S1 cortex at different developmental stages. This paper needs to be cited as it is highly relevant to this study.

Reyes and Sakmann (J. Neurosci. 1999) reported layer-specific differences in short-term plasticity in young sensorimotor cortex which disappeared as maturation progressed and short-term plasticity increased. In a previous study (Bornschein et al., Front. Syn. Neurosci. 2019) we also described a developmentally driven increase in short-term plasticity caused by the maturation of vesicle pools. In the present study we used mature animals and would therefore not expect layer-specific differences neither in S1 nor in PFC since the time course of postnatal maturation was described to be comparable in both neocortical circuits (Kroon et al., Sci. Rep. 2019).

We discussed this paper in the context of developmental changes in short-term plasticity.

“Short-term plasticity changes during postnatal development at different cortical PN connections without alterations in *p*_N_ (Reyes and Sakmann, 1999; Bornschein et al., 2019a). For L5PN-to-L5PN connections these differences were found to result from the maturation of an intermediate replenishment vesicle pool (Bornschein et al., 2019a). Such pool maturation may also underlie the elimination of layer-specific differences in short-term plasticity between L2/3-L5B and L5B-L5B synaptic connections that were evident in young rats but eliminated during the first weeks of postnatal development (Reyes and Sakmann, 1999). Since postnatal development follows the same time course in mouse PFC and S1 and occurs predominantly within the first two weeks after birth, significant layer-specific differences in PN synapses are unlikely in both areas in our experimental time window (Kroon et al., 2019). Consistently, we found similar PPRs at L2/3-L5PN synapses and L5PN-L5PN synapses in both areas, with facilitation in PFC and depression in S1, irrespective of the presynaptic PN synapse type.

(6) Regarding the point of loose or tight Ca^2+^ channel coupling: Could some of the differences result from differences in the presynaptic Ca^2+^ channel complement? Please comment.

This can be excluded. Ca_v_2.1 and Ca_v_2.2 are the main channels gating release at PN synapses. The gating kinetics of these channels are very similar and they only differ somewhat in their peak current amplitude (Bornschein et al., Cell Rep. 2019, Figure 4). Since the number of open channels is a fit parameter in our simulations there would only be an effect on the estimate of the number of channels gating release but not for the estimate of the coupling distance. This is all the more true since the EGTA effect depends on the diffusion distance rather than on the gating kinetics. These considerations will also hold for Ca_v_2.3 channels, which have slower closing kinetics, but anyway play only a very minor role for triggering release.
